# Development of bacterial resistance in Germany from 2008 to 2022 — major culprit pathogens, antibacterial drugs, and prescribing practices

**DOI:** 10.1007/s00210-024-03533-6

**Published:** 2024-10-23

**Authors:** Lilly Josephine Bindel, Roland Seifert

**Affiliations:** Institute of Pharmacology, Hannover Medical School, Hannover, D-30625 Germany

**Keywords:** Antimicrobial consumption, Antibiotic, Antibacterial drug, AMR, Antibacterial resistance, Antibiotic prescription, Germany, Arzneiverordnungsreport, Surveillance, Antibiotic Stewardship, Correlation, Rational prescribing behaviour, Irrational prescribing behaviour, Prediction

## Abstract

Rising bacterial resistance is a global threat, causing rising financial burdens on healthcare systems and endangering effective treatment of bacterial infections. To ensure the efficacy of antibacterial drugs, it is essential to identify the most dangerous pathogens and vulnerable antibacterial drugs. Previous research by our group suggested irrational outpatient prescribing practices in Germany, supporting a growing bacterial resistance. This study analyses developments and characteristics for the ten most prescribed antibacterial drugs in Germany from 2008 to 2022. Conclusions are based on the development of bacterial resistance levels and an analysis of correlations between pathogens. We identified cefuroxime axetil, sulfamethoxazole-trimethoprim and nitrofurantoin as the most problematic drugs. Particularly problematic pathogens include *E. faecalis*, *E. faecium*, *K. pneumoniae*, and *P. mirabilis*. Besides increasing bacterial resistance, they are characterised by a high proportion of significant positive correlations, indicating a high potential for mutually reinforcing resistance development. Alarmingly, most of the antibacterial drugs analysed showed a growing resistance to at least one of the analysed pathogens. In most cases, the best treatment option is threatened by increasing bacterial resistance. We also identified several differences between current bacterial resistance data and therapeutic guidelines. In aggregate, our findings support irrational prescribing behaviour and underscore the urgent need for improved prescribing practices to counter rising bacterial resistance in Germany. Moreover, therapeutic guidelines for bacterial infections, the “holy grail” of pharmacotherapy, must be updated more frequently.

**Supplementary Information:**

The online version contains supplementary material available at 10.1007/s00210-024-03533-6.

## Introduction

In recent years, “bacterial antimibrobial resistance (AMR) […] has emerged as one of the leading public health threats of the 21^st^ century”, being “a health problem whose magnitide is at least as large as major diseasus such as HIV and malaria, and potentially much larger” (Antimicrobial Resistance Collaborators 2022). Furthermore, it places a significant financial burden on the healthcare system (O’Neill 2014). In order to counter these serious developments, it is essential to identify the most problematic pathogens and the respective antibacterial drugs. Previous research from our group has demonstrated that financial considerations play a major role in antibacterial drug prescription in Germany, resulting in irrational drug prescription and increase in bacterial resistance (Bindel and Seifert 2024a, b, c).

Our present study aims to analyse trends and identify the most problematic developments within the ten most prescribed antibacterial drugs in outpatient settings in Germany between 2008 and 2022. Besides a characterisation of developments, we identified the potential for interference between pathogens by examining correlations between bacterial resistances. Our findings will help to take appropriate measures to promote rational prescribing behaviour in a targeted manner in order to counteract the development of rising bacterial resistance.

## Materials and methods

### Data collection of DDD prescriptions

The analysis of DDD prescriptions of antibacterial drugs is based on the Arzneiverordnungsreport (AVR, Drug prescription report), spanning from 2008 to 2022 (Schwabe and Paffrath 2010a, 2010b, 2011a, 2011b, 2012, 2013, 2014, 2015, 2016, 2017; Schwabe et al. 2018, 2019; Schwabe and Ludwig 2020; Ludwig et al. 2021, 2023, 2024). Since we focused on outpatient prescriptions, only the general chapter “Antibiotika und Chemotherapeutika” (antibiotics and chemotherapeutics) was considered. Therefore, specialised subchapters like urology, dermatology, and ophthalmology were excluded.

### Data collection of bacterial resistance

Bacterial resistance data was sourced from the Antibiotic Resistance Surveillance (ARS, https://ars.rki.de/) statistics provided by the Robert Koch Institute (RKI), covering the years since 2008 (RKI 2024).

Various options had to be selected. We chose the outpatient care area and included all material groups, regions, and specialisations. Given were the values for sensitive, intermediate, and resistant pathogens in per cent. We only took into account the percentage of resistant pathogens.

### Selection of drugs

The study focused exclusively on antibacterial drugs. We identified the top 15 most prescribed drugs in 2022: amoxicillin, cefuroxime axetil, doxycycline, amoxicillin-clavulanic acid, clindamycin, azithromycin, phenoxymethylpenicillin, sulfamethoxazole-trimethoprim, nitrofurantoin, ciprofloxacin, clarithromycin, cefaclor, cefpodoxime, pivmecillinam, and roxithromycin.

A lack of data availability for resistance statistics led to the exclusion of phenoxymethylpenicillin and the drugs ranked 12–15. The final list comprised ten drugs: amoxicillin, cefuroxime axetil, doxycycline, amoxicillin-clavulanic acid, clindamycin, azithromycin, sulfamethoxazole-trimethoprim, nitrofurantoin, ciprofloxacin, and clarithromycin.

### Analysis of correlations

Correlation analysis of bacterial resistance between analysed pathogens within each antibacterial drug was conducted using SPSS, with a bivariate correlation analysis employing the Pearson correlation coefficient. The coefficient of determination (*R*^2^) was calculated manually. Data processing and visualization were performed using SPSS and Excel.

During the analysis, we emphasized three aspects of the correlations: significance, direction (positive vs. negative), and strength. The Pearson coefficient indicates whether there is a linear relationship between the two variables. The correlation coefficient ranges from −1 to +1. A positive coefficient indicates that both variables influence each other in the same direction, while a negative coefficient indicates an inverse relationship. A value of 0 signifies no linear relationship, while a value of 1 indicates a very strong linear relationship with same proportions of growth (Mukaka 2012). We define a correlation above (+/−) 0.8 as strong, indicating a substantial influence between both factors. Values below 0.8 suggest a weaker relationship.

Besides the correlation coefficient, the significance of the correlation should be considered. The significance level indicates the extent to which the values can be generalized and considered reliable. Only significant values validate the correlation coefficient, allowing to draw conclusions. If there is non-significance, the values are only limited informative. A value with a significance level of 0.01 as well as 0.05 is determined to be considered significant.

The coefficient of determination (*R*^2^), calculated by squaring the Pearson correlation coefficient, indicates the proportion of the variance in the dependent variable that is predictable from the independent variable. Besides the significance, it can be used as an indicator whether the given correlation is valid.

In Figure 1, the methodical procedure is illustrated. Tables 1, 2, 3, 4, 5, and 6 and Figs. 2, 3, 4, and 5 show the most important results and conclusions of our study for the pharmacologist and physician. Supplemental Tables S1–S10 and supplemental Figs. S1–S15 as well as the text accompanying these items present a very detailed analysis that is more revelant for medical microbiologists.

**Fig. 1:**
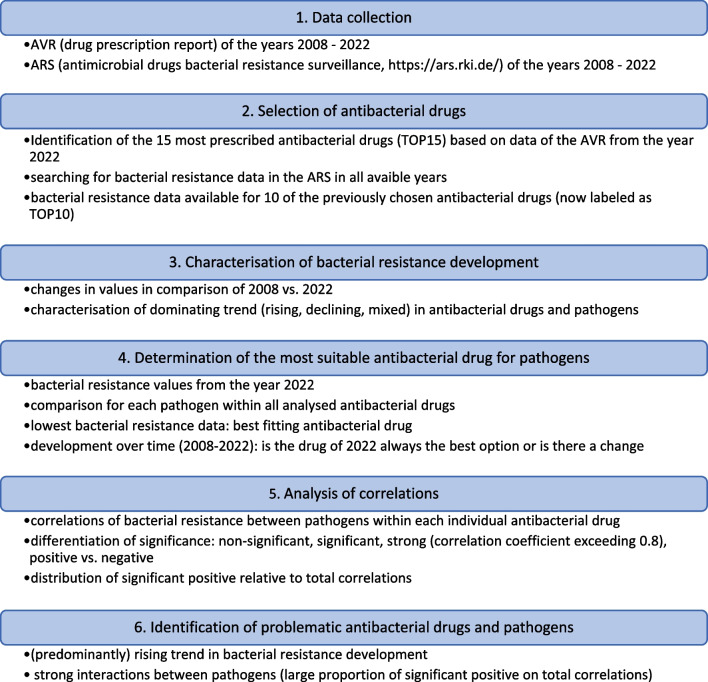
Methodical approach of the selection of antibacterial drugs, analysis of correlations between analysed pathogens, and the prediction of bacterial resistance development

## Results and discussion

### Development of bacterial resistance: characterisation of antibacterial drugs

It is essential to get an overview about developments of bacterial resistance of the most commonly used antibacterial drugs, so trends can be characterised and conclusions made. In the following, the development of bacterial resistance is characterized by comparing the first data point, starting from 2008, with the latest available data from 2022. Table 1 provides an overview of the trends for each antibacterial drug, while the curves for each antibacterial drug are depicted in Figs. S1–S10.

**Table 1:**
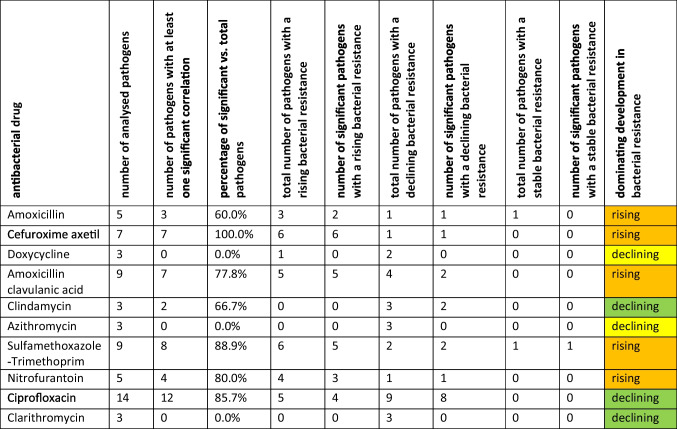
Overview about characteristics for antibacterial drugs with regard of correlations between pathogens and the development of bacterial resistance for analysed pathogens

As a conclusion, the dominating development is given. If it is defined as “rising” (orange colour), the majority of pathogens have seen a rising resistance. If it is defined as “declining” (yellow colour), the majority of analysed pathogens are declining, although there are also rising pathogens. If it is defined as “declining” (green colour), all analysed pathogens declined in terms of bacterial resistance

If bacterial resistance increases in the examined period, the trend is classified as rising. If it decreases, the trend is classified as declining. If there is no change in resistance, the trend is considered stable. These trends can be generalised for each drug by summarising the resistance trends across all analysed pathogens. The trends are further categorised into uniformly rising, uniformly declining, or mixed, with either rising or declining resistance dominating, which allows to assess problematic developments. Additionally, a short overview about correlations between pathogens is already given here for taking into account the potential of interactions between analysed pathogens.

A declining trend of bacterial resistance development in all pathogens is depicted for clindamycin (**3 of 3**), azithromycin (**3 of 3**), and clarithromycin (**3 of 3**). Notably, there are no significant correlations between pathogens for these three drugs (**0.0%**), which may be a hint that only minor interactions occur within susceptible pathogens. This favourable development may be attributed to the decreasing DDD prescriptions in recent years for all three drugs (Bindel and Seifert 2024a). Literature suggests that an increase in DDD prescriptions promotes the development of bacterial resistance (Abejew et al. 2024, Bell et al. 2014, Goossens 2009), which did not take place here.

A dominating decline in resistance, despite some rising and declining pathogens within a single drug, is evident for doxycycline (**2 of 3**) and ciprofloxacin (**9 of 14**). While only three pathogens were analysed for doxycycline, 14 pathogens were examined for ciprofloxacin. This disparity may explain why doxycycline has no significant pathogen correlations (**0.0%**), while ciprofloxacin shows significant correlations in the majority of examined pathogens (**85.7%**). Analysing the DDD prescription trends, both drugs have seen a decline in recent years, although they were once popular (Bindel and Seifert 2024a).

No antibacterial drug shows a uniformly rising resistance trend across all analysed pathogens. However, a rising trend is dominant in amoxicillin (**3 of 5**), cefuroxime axetil (**6 of 7**), amoxicillin clavulanic acid (**5 of 9**), sulfamethoxazole-trimethoprim (**6 of 9**), and nitrofurantoin (**4 of 5**). Notably, these drugs have a relatively large number of analysed pathogens and a high percentage of significant correlations between pathogens, ranging from 60.0 to 100.0%, indicating a vivid interaction between susceptible pathogens. When examining the development of DDD prescriptions, it is remarkable that the consumption of most of these drugs, except for sulfamethoxazole-trimethoprim, has shown an increasing trend in recent years (Bindel and Seifert 2024a). As a consequence, it can be assumed that the increase in consumption supported the rise in bacterial resistance (Abejew et al. 2024, Bell et al. 2014, Goossens 2009).

**Fig. 2:**
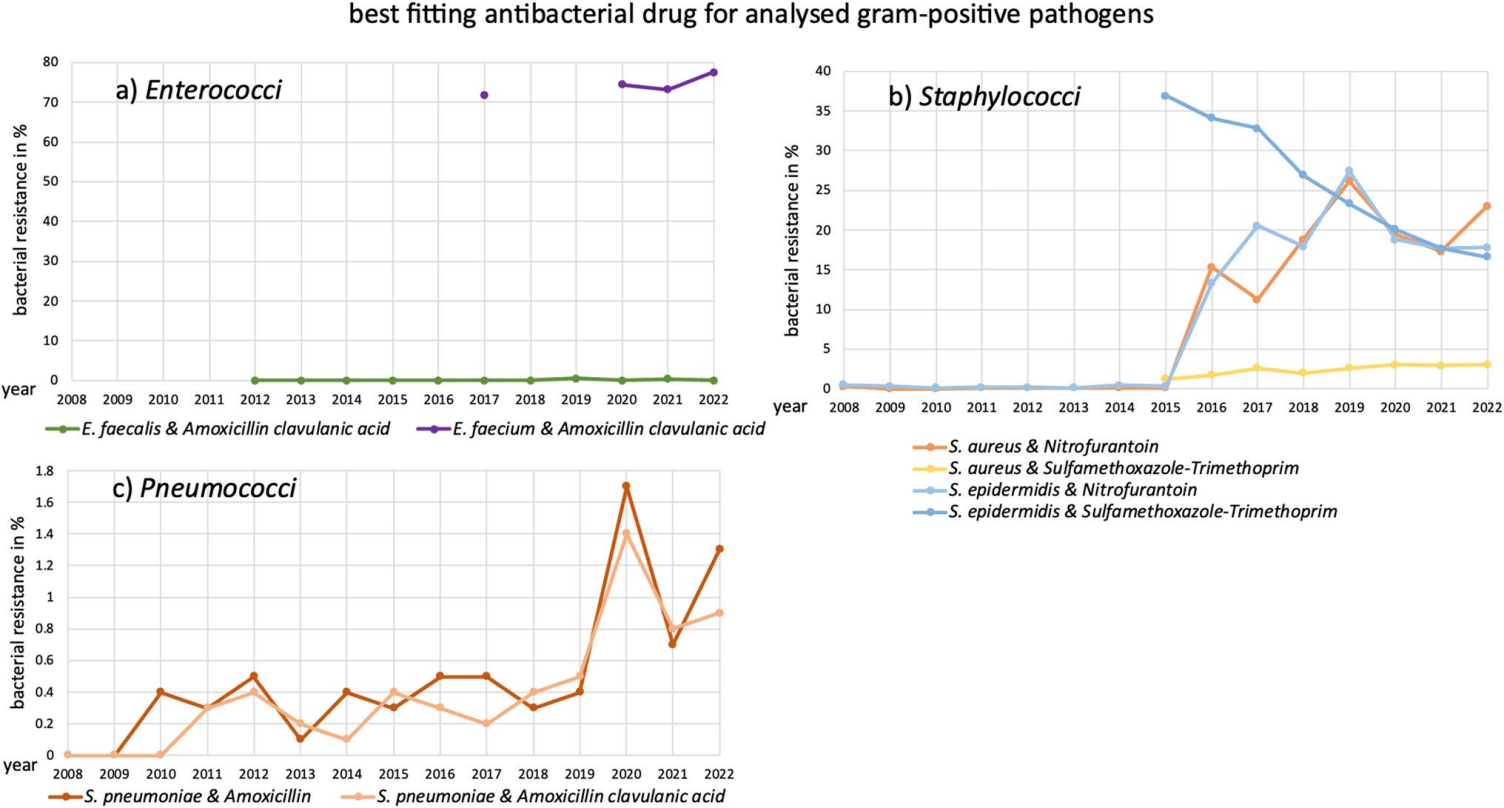
The best fitting antibacterial drug for treating an infection with the respective gram-positive pathogens (*Enterococci*, *Staphylococci*, *Pneumococci*) and its development of bacterial resistance in the analysed time period is depicted. If there was a change within the matching drug (including *S. aureus*, *S. epidermidis*, and *S. pneumoniae*), both antibacterial drugs and their development for the pathogen are exhibited, marked by similar colours

Drugs with broad indications, such as cefuroxime axetil and sulfamethoxazole-trimethoprim, often face a higher risk of developing bacterial resistance compared to drugs with narrower indications, like doxycycline, azithromycin, and clarithromycin. Decreasing resistance of a pathogen in the majority of analysed drugs does not necessarily translate to a reduction in resistance for the most efficient treatment against a particular pathogen. For instance, while *S. aureus* shows a declining trend in resistance for several drugs, resistance continues to rise for its most effective treatment when considering the most suitable antibacterial drug (Table 2 and Fig. 2).

In summary, a concerning rise in bacterial resistance is depicted for amoxicillin, cefuroxime axetil, amoxicillin clavulanic acid, sulfamethoxazole-trimethoprim, and nitrofurantoin. A mixed trend is depicted for doxycycline and ciprofloxacin. Fortunately, resistance to all pathogens is declining for clindamycin, azithromycin, and clarithromycin. Nevertheless, the majority of drugs analysed have at least one pathogen with rising bacterial resistance.

### Development of bacterial resistance: characterisation of pathogens

In addition to generalising trends for antibacterial drugs, the development of bacterial resistance for each pathogen can also be characterised as rising, declining, or mixed. This comparison as well as the assessment of potential interactions can be performed similarly to that for antibacterial drugs. Table 2 provides an overview of these trends.

**Table 2:**
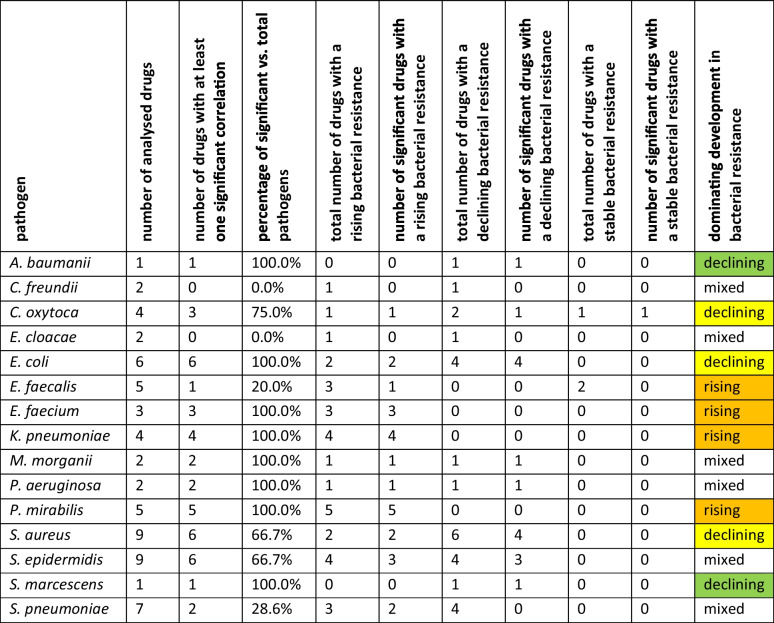
Overview about characteristics for pathogens with regard of correlations between pathogens and the development of bacterial resistance for analysed pathogens

As a conclusion, the dominating development is given. If it is defined as “rising” (orange colour), the majority of pathogens have seen a rising resistance. If it is defined as “declining” (yellow color), the majority of analysed pathogens are declining, although there are also rising pathogens. If it is defined as “declining” (green colour), all analysed pathogens declined in terms of bacterial resistance. If it is defined as “mixed” (no colour), rising and declining developments of bacterial resistance are balanced

A consistent rise in bacterial resistance across all analysed drugs is observed in *E. faecium*, *K. pneumoniae*, and *P. mirabilis*. A predominantly rising trend, with only a few instances of declining or stable resistance, is noted for *C. oxytoca* and *E. faecalis*. Notably, all these pathogens, except *E. faecalis*, exhibit significant correlations within each analysed drug (**100.0%** compared to **20.0%** for *E. faecalis*). This may be a hint for a high potential for interactions between different strains of pathogens.

A uniform decline in resistance is only observed in *A. baumanii* and *S. marcescens*, though this finding is limited by the low number of analysed antibacterial drugs (**1**). For *C. oxytoca*, *E. coli*, and *S. aureus*, declining bacterial resistance is dominant, although some drugs still show increasing resistance. Importantly, the proportion of significant correlations among total pathogens is very high for these bacteria, ranging from 66.7 to 100.0%. This might be reasoned by a large number of analysed antibacterial drugs. Nevertheless, it is a hint for problematic behaviour, since there is given a potential for mutual influence.

Mixed trends, which cannot be definitively characterized as either rising or declining, are observed in *C. freundii*, *E. cloacae*, *M. morganii*, *P. aeruginosa*, *S. epidermidis*, and *S. pneumoniae*. For these pathogens, the balance between rising and declining or stable trends is relatively even, making it difficult to determine a clear trend. The percentage of significant correlations relative to the total number of drugs varies widely. While *C. freundii* and *E. cloacae* have no significant correlations (**0.0%**), *M. morganii* and *P. aeruginosa* show a high percentage (**100.0%**), though based on data from only two analysed pathogens. *S. pneumoniae* (**28.6%**) and *S. epidermidis* (**66.7%**) fall somewhere in between.

A division between homogenous and heterogenous development is useful to categorize trends in bacterial resistance. Homogenous trends can be recognised by a large number as well as a high proportion of significant correlations, and can include either fortunately or problematic developments. A large proportion of significant positive on total correlations can be a hint to problematic behaviour. A homogenous trend is depicted for *A. baumanii*, *E. faecalis*, *E. faecium*, *K. pneumoniae*, *P. mirabilis*, and *S. marcescens*. Except *A. baumanii* and *S. marcescens* with a uniformly declining bacterial resistance, all other homogenous pathogens exhibit only rising bacterial resistance. This marks them as pathogens with a problematic susceptibility. In contrast, pathogens with heterogenous trends are more difficult to characterise and to assess whether effective treatment is at risk. This includes *E. coli*, *C. freundii*, *E. cloacae*, *C. oyxtoca*, *M. morganii*, *P. aeruginosa*, *S. aureus*, *S. epidermidis*, and *S. pneumoniae*.

**Fig. 3:**
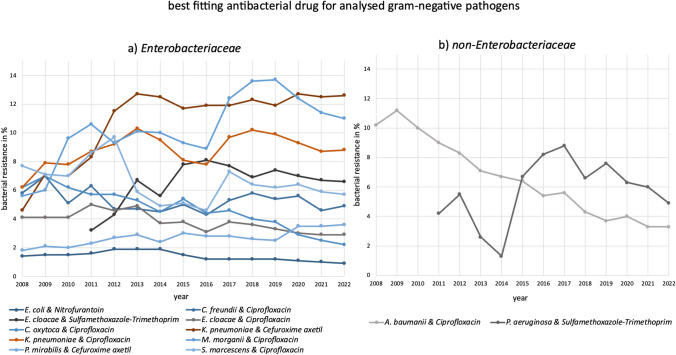
The best fitting antibacterial drug for treating an infection with the respective gram-negative pathogens (*Enterobacteriaceae*, *non-Enterobacteriaceae*) and its development of bacterial resistance in the analysed time period is depicted. If there was a change within the matching drug (including *E. cloacae* and *K. pneumoniae*), both antibacterial drugs and their development for the pathogen are exhibited, marked by similar colours

A differentiation has to be made between a general trend of bacterial resistance development for antibacterial drugs and the susceptibility for the most effective treatment (see the chapter “Most suitable antibacterial drug for analysed pathogens” section). Decreasing resistance in the majority of analysed drugs does not necessarily translate to a reduction in resistance for the most effective treatment against a particular pathogen. For instance, while *S. aureus* shows a declining trend in resistance for several drugs, resistance continues to rise for its most suitable treatment (see Table 2 and Fig. 3). This results in a higher risk of treatment failure.

In summary, a concerning rise in bacterial resistance for all analysed antibacterial drugs is depicted for *E. faecalis, E. faecium, K. pneumoniae* and *P. mirabilis*, indicating a homogenous, particularly concerning trend. Besides this, there are many pathogens with a mixed trend, but only *A. baumanii* and *S. marcescens* exhibit uniform decline. Literature research confirms growing AMR for the most conspicious pathogens *E. faecalis*, *E. faecium*, *K. pneumoniae*, and *P. mirabilis* (Guan et al. 2024; ECDC 2023; Al-Qurashi et al. 2022). This is particularly alarming for highly dangerous pathogens like *K. pneumoniae*, which are among the leading deathly pathogens globally (Antimicrobial Resistance Collaborators 2022). In general, any increase in bacterial resistance poses a potential threat to the future effectiveness of bacterial infection treatments, especially since nearly all pathogens exhibit growing bacterial resistances.

### Most suitable antibacterial drug for analysed pathogens

**Fig. 4:**
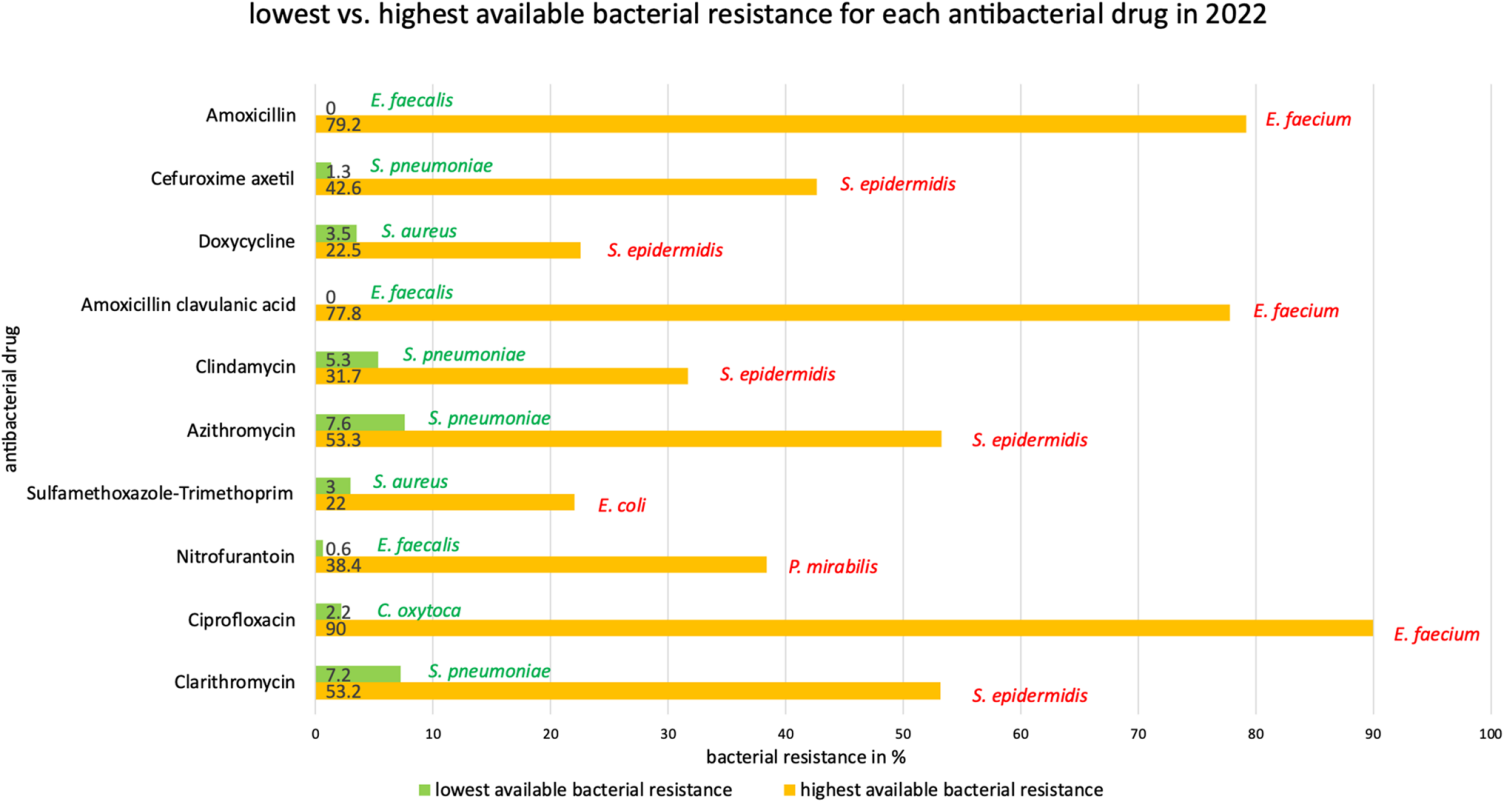
Overview of the lowest vs. highest available bacterial resistance in 2022, sorted by antibacterial drugs. The analysis includes the TOP10 antibacterial drugs and their analysed pathogens. For each antibacterial drug, the pathogen with the lowest (green) and highest (red) bacterial resistance is shown. Importantly, the pathogen with the lowest bacterial resistance does not indicate that the antibacterial drug is the best choice, as the pathogen may have a lower bacterial resistance within another antibacterial drug (e.g. *E. faecalis, S. pneumoniae*, *S. aureus*)

**Fig. 5:**
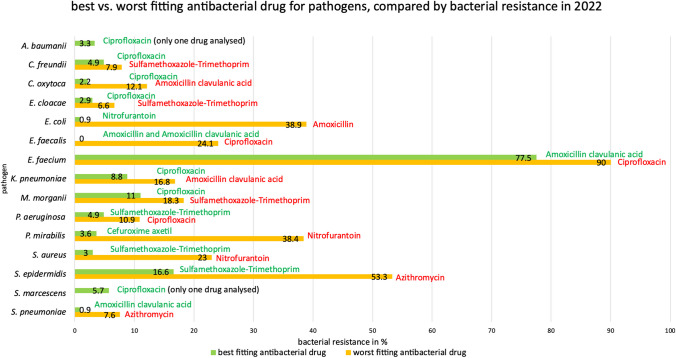
Overview of the best vs. worst antibacterial drug to treat a bacterial infection with the respective pathogen. The analysis is based on bacterial resistance rates in 2022 and includes the TOP10 antibacterial drugs and their analysed pathogens. For each pathogen, the bacterial resistance of the best fitting (green colour) and worst fitting (red colour) antibacterial drug is shown. The drug with the lowest bacterial resistance is also the best fitting option for each pathogen in terms of bacterial resistance. In some cases, only one antibacterial drug was analysed, making a comparison impossible; this drug is considered the best option because it is the only one of the TOP10 that has an indication and was therefore analysed in the ARS (RKI 2024)

In an outpatient setting, it is crucial to determine the most effective antibacterial drug for treating infections caused by specific pathogens. While it is ideal to perform an antibacteriogram for precise guidance, when this is not possible, the physician must select the drug most likely to be effective based on historical resistance patterns. This section outlines the antibacterial drug which is the best treatment option for a certain pathogen, based on the lowest bacterial resistance among the tested drugs. Figures 2 and 3 depict the trends of analysed pathogens, sorted by gram-positive and gram-negative strains. More detailed information, including bacterial resistance rates, can be found in Figs. 4 and 5 and Figs. S11–S15.

Doxycycline, clindamycin, azithromycin, and clarithromycin are not the optimal choices for any of the analysed pathogens in terms of low bacterial resistance. This may be due to the limited number of pathogens analysed for these drugs, or because alternative antibacterial drugs consistently exhibited lower resistance.

Amoxicillin is the best option for *E. faecalis*, along with amoxicillin clavulanic acid. There was an increase in bacterial resistance over the years analysed (0.6% bacterial resistance in 2021), but this fell back to 0.0% in 2022. Guidelines list amoxicillin as one of the first choices for the treatment of *E. faecalis* (EUCAST 2010; Delgado et al. 2023; AWMF 2024; PEG 2019).

Cefuroxime axetil is the best choice for *P. mirabilis*, although an increase in bacterial resistance has been observed. Nevertheless, it is still recommended as the first choice in German guidelines (AWMF 2017; PEG 2019). From 2008 to 2011, cefuroxime axetil also had the lowest resistance rates for *K. pneumoniae*, but it was replaced by ciprofloxacin in 2012 due to rising resistance.

Amoxicillin clavulanic acid is the most effective option for *E. faecalis*, *E. faecium*, and *S. pneumoniae*, despite rising bacterial resistance in all three pathogens. Data for *E. faecalis* and *E. faecium* were unavailable at the start of the period, but since it became available, amoxicillin clavulanic acid has shown the lowest resistance and is therefore the best choice. For *S. pneumoniae*, amoxicillin was sometimes a better option, but by 2022, amoxicillin clavulanic acid had the lowest resistance rates. In guidelines, it is listed as a suitable option for *E. faecalis* and *S. pneumoniae* (Delgado et al. 2023; AWMF 2024a; PEG 2019). In contrast, it is not recommened for *E. faecium* because of high bacterial resistance rates (PEG 2019).

Sulfamethoxazole-trimethoprim is the most suitable treatment for *P. aeruginosa*, *S. aureus*, and *S. epidermidis*. Between 2011 and 2012, it was also the best option for *E. cloacae* but was later replaced by ciprofloxacin. While bacterial resistance is rising in *S. aureus*, *E. cloacae*, and *P. aeruginosa*, it is declining in *S. epidermidis*. Sulfamethoxazole-trimethoprim was not the first choice for any pathogen in 2008, partly because bacterial resistance data only became available in 2011. It became the best option for *E. cloacae* and *P. aeruginosa* in 2011, for *S. epidermidis* in 2012, and for *S. aureus* in 2019. Nevertheless, it is not recommended in the latest guidelines for any of the pathogens (PEG 2019; AWMF 2017, 2019a, 2023).

Nitrofurantoin is the best option for *E. coli*, with bacterial resistance fortunately declining. In guidelines, it is mentioned as a suitable treatment option (AWMF 2024). It also had the lowest resistance in *S. aureus* (from 2008 to 2015) and *S. epidermidis* (from 2008 to 2018, and in 2020), but rising resistance led to it being replaced by sulfamethoxazole-trimethoprim for both pathogens.

Ciprofloxacin is the most suitable drug for *A. baumannii*, *C. freundii*, *C. oxytoca*, *E. cloacae*, and *S. marcescens*, with bacterial resistance decreasing in all these pathogens. For a brief period between 2011 and 2012, sulfamethoxazole-trimethoprim was the best option for *E. cloacae*, but ciprofloxacin quickly regained its position. Literature research suggests that ciprofloxacin is not recommended as a first-line therapy for any of the pathogens mentioned (AWMF 2017, 2018, 2024a; Abdul-Mutakabbir et al. 2021; Ewig et al. 2021; PEG 2019; PEG 2016).

In pathogens, increasing bacterial resistance is observed for *E. faecalis*, *E. faecium*, *K. pneumoniae*, *M. morganii*, *P. aeruginosa*, *S. aureus*, and *S. pneumoniae*. While resistance in *S. epidermidis* has increased when regarding the entire period, it has decreased in recent years for sulfamethoxazole-trimethoprim but 2022 is still higher than 2008. Pathogens showing a decrease in bacterial resistance include *E. coli*, *A. baumannii*, *C. freundii*, *E. cloacae*, *C. oxytoca*, and *S. marcescens*. Changes in the most favourable antibacterial drug occurred for *E. cloacae*, *K. pneumoniae*, *S. aureus*, *S. epidermidis*, and *S. pneumoniae*. For other pathogens, a single antibacterial drug remained the best option throughout the observed period. There is no difference in behaviour recognizable between positive and negative gram strains.

No antibacterial drug exhibited falling bacterial resistance in all pathogens for the best treatment. Only rising bacterial resistance is depicted for amoxicillin in one case (*S. pneumoniae*), for cefuroxime axetil in two (*K. pneumoniae*, *P. mirabilis*), and for amoxicillin clavulanic acid in three cases (*E. faecalis*, *E. faecium*, *S. pneumoniae*). For these three drugs, the rising trend led to the removal of the best treatment option in certain pathogens, including *K. pneumoniae* and *S. pneumoniae*.

Both rising and falling bacterial resistance are exhibited for sulfamethoxazole-trimethoprim, nitrofurantoin, and ciprofloxacin. While the number of rising pathogens is dominating in sulfamethoxazole-trimethoprim (three rising: *E. cloacae*, *P. aeruginosa*, *S. aureus*; one falling: *S. epidermidis*) and nitrofurantoin (two rising: *S. aureus*, *S. epidermidis*; one falling: *E. coli*), more falling than rising bacterial resistance is depicted for ciprofloxacin (two rising: *P. aeruginosa*, *S. aureus*; five falling: *A. baumanii*, *C. freundii*, *E. cloacae*, *C. oxytoca*, *S. epidermidis*).

Guidelines indicate that, for some of the TOP15 antibacterial drugs, the first-line treatment choices do not align with the analysis of low bacterial resistance among the pathogens studied. This discrepancy can be attributed to several factors, e.g. the consideration of adverse effects. Moreover, guidelines are not published every year, but here, we considered the most current data available. Since resistance changes over time, guidelines can become outdated rapidly. In Tables 5 and 6, first-line recommendations of current guidelines in Germany are compared with our findings. Additionally, the analysis was restricted to specific pathogens and the TOP15 antibacterial drugs, thereby excluding more specialized and effective medications that are recommended only for particular pathogens in specific cases. This indicates that some pathogens are not effectively treated by the most prescribed drugs, also recognisable from high resistance rates. Nevertheless, the analysis provides a valuable assessment of treatment options. Accurate pathogen identification therefore plays an important role in the targeted treatment of infections.

In summary, the optimal treatment for seven pathogens is threathened by rising bacterial resistance for the best fitting pathogen, while a decrease is only depicted for six pathogens. All antibacterial drugs have increasing bacterial resistance in at least one pathogen for which they are the best treatment option. Beside this, rising bacterial resistance dominates over declining values. Literature research confirms an increasingly resistance to current treatment options (Yee et al. 2020), besides a general trend of growing AMR (WHO 2022). This is a very worrying development that threatens the effective treatment of the particular bacterial infection.

### Interactions between pathogens

A correlation analysis was performed to identify and characterise relationships and interactions between pathogens. Therefore, the bacterial resistance of analysed pathogens was compared for each antibacterial drug. To evaluate the potential of mutually reinforcing resistance development for antibacterial drugs and pathogens, several characteristics can be considered. Key factors include the total number of significant correlations between pathogens, the number of significant positive correlations, and the proportion of positive correlations relative to total correlations. Tables 3 and 4 provide an overview about the considered characteristics, while Tables S1–10 exhibit detailed information about all correlations.

**Table 3:** Summary for antibacterial drugs about the characteristics in the correlations between pathogens within each individual drug

Ranking	Antibacterial drug	Number of total correlations	Number of significant correlations	Number of significant positive correlations	Proportion of significant positive vs. total correlations
1	Amoxicillin	10	2	1	10.0%
2	Cefuroxime axetil	21	13	8	38.1%
3	Doxycycline	3	0	0	0.0%
4	Amoxicillin clavulanic acid	36	7	4	11.1%
5	Clindamycin	3	1	1	33.3%
6	Azithromycin	3	0	0	0.0%
7	Sulfamethoxazole-trimethoprim	36	17	12	33.3%
8	Nitrofurantoin	10	6	3	30.0%
9	Ciprofloxacin	91	25	19	20.9%
10	Clarithromycin	3	0	0	0.0%

**Table 4:** Summary for pathogens about the characteristics in the correlations between pathogens for all analysed antibacterial drugs

Pathogen	Gram strain	Number of total correlations	Number of significant correlations	Number of significant positive correlations	Proportion of significant positive vs. total correlations
*A. baumanii*	-	13	8	5	38.5%
*C. freundii*	-	21	0	0	0.0%
*C. oxytoca*	-	35	13	9	25.7%
*E. cloacae*	-	21	8	7	33.3%
*E. coli*	-	43	21	13	30.2%
*E. faecalis*	+	17	2	2	11.8%
*E. faecium*	+	25	1	0	0.0%
*K. pneumoniae*	-	35	14	12	34.3%
*M. morganii*	-	21	12	8	38.1%
*P. aeruginosa*	-	21	3	3	14.3%
*P. mirabilis*	-	35	16	12	34.3%
*S. aureus*	+	47	21	9	19.1%
*S. epidermidis*	+	47	17	13	27.7%
*S. marcescens*	-	13	2	2	15.4%
*S. pneumoniae*	+	27	7	4	14.8%

Pathogens were divided by positive (+) or negative (−) gram strain

In the correlations between pathogens, only non-significant correlations were found in doxycycline, azithromycin, and clarithromycin. All other drugs have significant as well as strong correlations, defined as a correlation coefficient exceeding 0.8, between their analysed pathogens. More detailed information for each antibacterial drug can be found in the supplement.

When differentiating between gram-negative and gram-positive pathogens, there are no differences recognizable (see Table 4 and Tables S1–S10). Pathogens can have a significant correlation with the same as well as with the other gram strain. This observation may be alarming as it allows for the possibility of cross-resistance between the two groups.

To assess the extent of similar trends in bacterial resistance, the number of significant positive on total correlations can be considered. Positive correlations are particularly relevant for identifying problematic developments, as they suggest mutual reinforcement of resistance trends. A high proportion may indicate potential cross-resistance or at least a mutual relationship between pathogens that could reinforce the development of bacterial resistance. Drugs and pathogens can be made comparable by examining the ratio instead of the total numbers, since a large number of analysed pathogens or drugs offers the possibility for a higher number of significant correlations. Further explanations about the number of signficant correlations are provided in the supplemental data.

The number of significant positive correlations ranges in antibacterial drugs from 0 for doxycycline, azithromycin, and clarithromycin to 19 for ciprofloxacin. The distribution of high and low numbers closely mirrors the total number of significant correlations. Proportions of significant positive correlations relative to total correlations range from 0.0% for doxycycline, azithromycin, and clarithromycin to 38.1% for cefuroxime axetil. Drugs with proportions exceeding 30.0% include cefuroxime axetil (**38.1%**), sulfamethoxazole-trimethoprim (**33.3%**), clindamycin (**33.3%**), and nitrofurantoin (**30.0%**). More varying trends are observed for ciprofloxacin (**20.9%**), amoxicillin clavulanic acid (**11.1%**), and amoxicillin (**10.0%**).

For pathogens, the number of significant positive correlations ranges from 0 for *C. freundii* and *E. faecium* to 13 for *E. coli* and *S. epidermidis*. The distribution is analogous to the total number of significant correlations. Notably, *E. faecium* exhibits no significant positive correlations, despite having one significant correlation overall, and *S. aureus* shows a relatively small number of significant positive correlations despite a high total of significant correlations. The proportion of significant positive correlations relative to total correlations ranges from 38.5% for *A. baumannii* to 0.0% for *C. freundii* and *E. faecium*. Generally, these shares are distributed continuously. Pathogens with proportions exceeding 30.0% include *A. baumannii* (**38.5%**), *M. morganii* (**38.1%**), *K. pneumoniae* (**34.3%**), *P. mirabilis* (**34.3%**), *E. cloacae* (**33.3%**), and *E. coli* (**30.2%**). *S. epidermidis* (**27.7%**) and *C. oxytoca* (**25.7%**) exhibit shares between 20.0 and 30.0%. Pathogens with proportions under 20.0% include *S. aureus* (**19.1%**), *S. marcescens* (**15.4%**), *S. pneumoniae* (**14.8%**), *P. aeruginosa* (**14.3%**), and *E. faecalis* (**11.8%**).

One can characterize an antibacterial drug or pathogen as tending to be influenced or independent. A high proportion indicates that pathogens influence each other similarly in resistance development, while a lower proportion suggests less coherence. The four antibacterial drugs with an exceptionally high proportion exhibit similar growth in bacterial resistance across analysed pathogens, except clindamycin with declining bacterial resistance trends. Importantly, a high number and proportion of positive correlations do not necessarily imply a rise in bacterial resistance, as a uniform decrease could also yield high values.

It is concerning that for four antibacterial drugs around one-third of the pathogens significantly share and influence their resistance development. This includes cefuroxime axetil, clindamycin, sulfamethoxazole-trimethoprim, nitrofurantoin, *A. baumanii*, *E. cloacae*, *E. coli*, *K. pneumoniae*, *M. morganii*, and *P. mirabilis.* Literature research confirms a potential for cross-resistance for mentioned pathogens (Japoni et al. 2011; Liu et al. 2016; Sanchez et al. 2013; Cherny et al. 2023; Annavajhala et al. 2019). Besides this, only two pathogens, *C. freundii* and *E. faecium*, are entirely uninfluenced by others, while the remaining 14 pathogens exhibit significant relationships with about one-third of the others, even with the other gram strain. These concerning features exhibited by these pathogens indicate a significant risk for rapid resistance development (Semenec et al. 2023).

### Particularly problematic antibacterial drugs and pathogens

While any increase in bacterial resistance poses a potential threat to the future effectiveness of bacterial infection treatments, it is crucial to identify the most problematic developments. This final summary synthesizes previously examined criteria, emphasizing the most notable results with a distinct and significant share. An assessment is then made based on the development of bacterial resistance within specific antibacterial drugs and pathogens.

When having a look at the development of bacterial resistance, a rise is predominant in the antibacterial drugs amoxicillin (**3 of 5**), cefuroxime axetil (**6 of 7**), amoxicillin clavulanic acid (**5 of 9**), sulfamethoxazole-trimethoprim (**6 of 9**), and nitrofurantoin (**4 of 5**) (Table 1 and Figs. S1, S2, S4, S7, S8). Among pathogens, a consistent increase in bacterial resistance across all analysed drugs is evident for *E. faecalis* (**3 of 5**), *E. faecium* (**3 of 3**), *K. pneumoniae* (**4 of 4**), and *P. mirabilis* (**5 of 5**) (Table 2). In terms of the best treatment options, bacterial resistance is increasing in all four pathogens mentioned, while most of the problematic antibacterial drugs are not the first indication, and if they are, the pathogens are also increasing dominantly (Figs. 2 and 3).

With regard to potential interaction between pathogens, exceptionally high numbers of significant positive correlations are depicted for ciprofloxacin (**19**), sulfamethoxazole-trimethoprim (**12**), *E. coli* (**13**), *S. epidermidis* (**13**), *K. pneumoniae* (**12**), and *P. mirabilis* (**12**) (Tables 3 and 4). Outstanding proportions of positive significant correlations relative to total correlations are noted for cefuroxime axetil (**38.1%**), clindamycin (**33.3%**), sulfamethoxazole-trimethoprim (**33.3%**), nitrofurantoin (**30.0%**), *A. baumannii* (**38.5%**), *M. morganii* (**38.1%**), *K. pneumoniae* (**34.3%**), and *P. mirabilis* (**34.3%**). There are significant correlations between gram-positive and gram-negative strains in nearly all antibacterial drugs and pathogens mentioned before. The only exception is clindamycin, since only gram-positive pathogens were analysed.

Antibacterial drugs and pathogens with predominantly rising bacterial resistance are often prominent in high numbers and proportions (see Tables 1, 2, 3, 4). A consistent trend, whether increasing or decreasing, appears to go hand in hand with high proportions across various metrics. Notably, trends of rising resistance exhibit a greater number of outstanding categories than those of decreasing resistance. The prevalence of multiple outstanding categories within rising resistance trends is particularly concerning as it indicates properties that have an unfavourable effect on the development of resistance.

In summary, the most concerning antibacterial drugs are cefuroxime axetil, nitrofurantoin, and sulfamethoxazole-trimethoprim. Among pathogens, *K. pneumoniae* and *P. mirabilis* are particularly notable for their serious characteristics. Besides a problematic development of bacterial resistance, they are exhibiting large proportions within examined data. Additionally, increased monitoring is necessary due to growing bacterial resistance for amoxicillin, amoxicillin clavulanic acid by *E. faecalis* and *E. faecium*. Literature research confirms a growing bacterial resistance for the identified antibacterial drugs and pathogens (Guan et al. 2024; ECDC 2023; Al-Qurashi et al. 2022). Generally, any increase in bacterial resistance should prompt further investigation into problematic behaviours within the respective antibacterial drug or pathogen.

## Limitations

The analysis in this study is based on data from the Arzneiverordnungsreport. As only outpatient prescriptions of the GKV system are included, no assessment can be made regarding prescriptions in hospitals or via private health insurance. As the data are based on developments in Germany, they are not directly transferable to other countries. No differentiation was made according to age or region.

Data collection for DDD prescriptions depended on the design of the chapter under consideration. Changes in the structure of the sections over the years led to a bias for clindamycin in 2012 (Schwabe and Paffrath 2013). The ARS criteria for analysing only indicated pathogens could exclude strong correlations, as a high increase in bacterial resistance might lead to the exclusion of the antibacterial drug in question. Both factors could lead to an under- or overestimation of the correlations in question.

Generalisations are restricted by the limited number of examined antibacterial drugs, pathogens, and factors considered. Data on bacterial resistance are only available for a short period of time compared to the available data on DDD prescriptions. Developments in bacterial resistance prior to 2008 were not analysed, which may lead to less accurate correlations. There may be other factors influencing the outcomes studied that were not included in the analysis.

Certain criteria were set for the statistical methods used. Between the choice of statistical procedures, the value for a significant correlation with a two-sided significance level of 0.01 and 0.05, as well as a determined strong correlation coefficient above (+/-) 0.8, was determined. Changing these parameters may lead to different conclusions.

## Conclusions

Several problematic antibacterial drugs and pathogens, respectively, were identified, including amoxicillin, cefuroxime axetil, amoxicillin clavulanic acid, sulfamethoxazole-trimethoprim, and nitrofurantoin, and *E. faecalis*, *E. faecium*, *K. pneumoniae*, and *P. mirabilis.* Literature research confirmed our findings (Markwart et al. 2019; Koppe et al. 2018; Werner et al. 2020; Girlich et al. 2020; PEG 2016). Besides a problematic development of bacterial resistance, they are exhibiting large proportions within examined data. In general, any increase in bacterial resistance is suspicious and needs to be addressed decisively. It is concerning that only three of the most prescribed antibacterial drugs show a decline in bacterial resistance, i.e. clindamycin, azithromycin, and clarithromycin, while the majority depicts a rise.

An examination of prescription trends for particularly problematic drugs such as cefuroxime axetil or amoxicillin clavulanic acid reveals a longer lasting increase in consumption (Bindel and Seifert 2024a). Since the significant increase in resistance has not been followed by a corresponding response, it confirms earlier assumptions that prescribing behaviour in Germany is not rational (Bindel and Seifert 2024c) and that treatment decisions are mostly driven by cost considerations rather than empirical evidence or the use of antibacteriograms (Bindel and Seifert 2024a, 2024b, 2024c). The warning signs indicate the presence of a structural issue, which requires the implementation of appropriate measures to counteract (Rödenbeck et al. 2023).

A comparison of the effectiveness of the best and worst antibacterial drugs against the pathogens reveals significant differences. An overview of the lowest and highest bacterial resistance for each of the TOP10 antibacterial drugs as well as for the analysed pathogens can be found in Figs. 4 and 5. While in the best case a low bacterial resistance is evident, the bacterial resistance can be much higher when choosing the worst fitting antibacterial drug, e.g. 0.9% vs. 38.9% for *E. coli*. The rate of bacterial resistance can vary greatly within an antibacterial drug between an appropriate and an inappropriate treatment, e.g. 0.0% vs. 79.2% in amoxicillin. It is therefore important to choose the most appropriate option and to use the latest data on bacterial resistance to ensure successful treatment of bacterial infections. The only way to accomplish this goal is to perform antibacteriograms routinely instead of using this methodology just in exceptional cases.

The correlation analysis revealed that strong interactions exist between pathogens in the majority of antibacterial drugs. In several cases, there is a significant positive correlation for about one-third of the pathogens analysed. This observation applies to all pathogens analysed and also exists between gram-positive and gram-negative strains. It can be assumed that mutual reinforcement and cross-resistance between pathogens will accelerate the growth of bacterial resistance (Lozano-Huntelman et al. 2020).

In terms of the overall situation, the results are alarming. AMR already cause a rising number of deaths globally and place a substantial financial burden on healthcare systems (Antimicrobial Resistance Collaborators 2022; O’Neill 2014). If this trend continues unattended, the effective treatment of bacterial infections will be seriously compromised, leading to a large number of treatment failures and deaths, even in Germany. In this context, it seems counterproductive to focus on the DDD costs of an antibacterial drug, although the resulting costs of non-rational prescribing practices are much higher. It is therefore imperative to quickly create the conditions for rational use, e.g. supported by price adjustments or usage restrictions. These measures have to be implemented quickly to preserve effectiveness until further measures, such as the development of new drugs and treatment options (Dance 2024), take effect.

### Further perspectives

Further research is needed to confirm our findings and to expand the analysis to include more antibacterial drugs and other drug classes, as well as more pathogens and time periods. Extending the study to other countries and comparing it with other countries will also provide a broader international perspective on the issue of increasing bacterial resistance.

In addition to an analysis of the current situation, a forecast of future developments would be useful to assess trends within antibacterial drugs and pathogens. This would provide an outlook on possible scenarios in order to prepare for them. For some pathogens, forecasts are already available (Rolff et al. 2024), but an analysis of the general trends or of the specific trends for Germany has not yet been carried out.

### Take-home messages

Our study clearly shows that bacterial resistance is increasing, urging for immediate measures. To preserve the effectiveness of antibacterial drugs, a rational prescribing practice has to be implemented:

1. **Use sparingly:** Antibacterial drugs are only effective against bacterial infections. Many viral infections are unnecessarily treated with antibacterial drugs in outpatient settings (Butler et al. 2010). Strict indications are therefore needed.
2. **Targeted use:** An antibacteriogram is strongly recommended before starting treatment with an antibacterial drug. This will ensure that an effective and specific antibacterial drug is used to prevent treatment failure.
3. **Know the current resistance situation:** When prescribing an antibacterial drug, it is important to be aware of the latest guidelines and best treatment options. Current data on bacterial resistance can be found online at the RKI (https://ars.rki.de/).
4. **Ignore the cost for your sick patient:** Although it may seem economical to prescribe a low-priced drug, the resulting cost of ineffective treatment is much higher. Therefore, the most appropriate drug must be chosen, regardless of DDD costs.
5. **Choose the drug wisely:** This study provides a rational basis for antibacterial drug use in outpatient settings.
6. **Stay up-to-date:** This study provides a template how up-to-date rational selection of the best antibacterial drug can be accomplished on the basis of publicly availalable data provided by the RKI and AVR. Guidelines can become outdated quickly to changed bacterial resistance (Tables 5 and 6).

**Table 5:** Comparison of treatment recommendations for antibacterial drugs by German guidelines and results for the TOP10 due to the lowest bacterial resistance

Pathogen	Recommended antibacterial drug, analysis based on bacterial resistance for TOP10 (status 2022)	Bacterial resistance 2022	Assessment of guideline with regard to first-choice treatment	Recommended antibacterial drug of guideline	Actuality of guideline
*A. baumanii*	*Ciprofloxacin*	3.3%	Not recommended (PEG 2019; AWMF 2017; AWMF 2024a)	Colistin, tigecyclin, sulbactam, sulfamethoxazole-trimethoprim	2018, 2017, 2024
*C. freundii*	*Ciprofloxacin*	4.9%	Not recommended (PEG 2019; AWMF 2024a; AWMF 2017)	Carbapeneme, piperacillin/tazocatam, cefepim	2018, 2024, 2016
*C. oxytoca*	*Ciprofloxacin*	2.2%	Not recommended (PEG 2019; PEG 2016; AWMF 2018)	Carbapeneme, piperacillin/tazocatam, cefepim	2018, 2016
*E. cloacae*	*Ciprofloxacin*	2.9%	Not recommended (PEG 2019; AWMF 2024a; PEG 2016)	Carbapeneme, piperacillin/tazocatam, cefepim	2010, 2018, 2024, 2016
*E. coli*	**Nitrofurantoin**	0.9%	recommended (AWMF 2024a)	Nitrofurantoin	2024
*E. faecalis*	**Amoxicillin, amoxicillin clavulanic acid**	0.0%	Recommended (Delgado et al. 2023; AWMF 2024a; PEG 2019)	Amoxicillin, amoxicillin clavulanic acid (alternative)	2023, 2024, 2018
*E. faecium*	*Amoxicillin clavulanic acid*	77.5%	Not recommended (PEG 2019)	Daptomycin, glykopeptides, linezolid, tigecyclin	2018, 2024
*K. pneumoniae*	*Ciprofloxacin*	8.8%	Not recommended (PEG 2016; PEG 2019; AWMF 2024a)	Carbapeneme, piperacillin/tazobactam, cephalosporins 3rd and 4th generation	2016, 2018, 2024
*M. morganii*	*Ciprofloxacin*	11.0%	Not recommended (PEG 2016; PEG 2019; AWMF 2024a)	Carbapeneme, cefepim	2016, 2018, 2024
*P. aeruginosa*	*Sulfamethoxazole-trimethoprim*	4.9%	Not recommended (PEG 2019; AWMF 2023)	Ceftazidim, piperacillin/tazobactam, ciprocloxacin	2018, 2023
*P. mirabilis*	**Cefuroxime axetil**	3.6%	Recommended (AWMF 2017; PEG 2019)	Cefuroxime axetil; ciprofloxacin (alternative)	2017, 2018
*S. aureus*	*Sulfamethoxazole-trimethoprim*	3.0%	Not recommended (PEG 2019; AWMF 2019a)	Cefazolin, cefuroxime axetil, amoxicillin, amoxicillin clavulanic acid, ampicillin/sulbactam	2018, 2019
*S. epidermidis*	*Sulfamethoxazole-trimethoprim*	16.6%	Not recommended (PEG 2019; AWMF 2019a)	Vancomycin, linezolid, daptomycin	2018, 2019
*S. marcescens*	*Ciprofloxacin*	5.7%	Not recommended (PEG 2016; PEG 2019; AWMF 2017)	Carbapeneme, cefepim	2016, 2018, 2017
*S. pneumoniae*	**Amoxicillin clavulanic acid**	0.9%	Recommended (Ewig et al. 2021; PEG 2019; AWMF 2024a)	Amoxicillin, amoxicillin clavulanic acid (alternative)	2021, 2018, 2024

Actual AWMF and PEG guidelines can be accessed online (https://www.awmf.org/leitlinien; https://www.p-e-g.org/leitlinienempfehlungen.html). Besides the findings of our analysis for the TOP10, the assessment of German guidelines is given. If both recommendations agree, the antibacterial drug is shown in bold, if not, it is shown in italics. Additionally, the first-choice treatment of the respective pathogen and the actuality of the guideline are included. In general, the guidelines mention that an antibacterial drug that is not the first choice, however, can be used for treatment if the susceptibility of the respective pathogen is ensured

**Table 6:** Comparison of treatment recommendations for pathogens by German guidelines and results for the TOP10 due to lowest bacterial resistance

Ranking	Antibacterial drug	Recommened pathogen, analysis based on lowest bacterial resistance for TOP10 (status 2022)	Bacterial resistance 2022	First-choice treatment for pathogens by German guidelines	Reference
1	Amoxicillin	***E. faecalis***	0.0%	*S. pneumoniae*, *E. faecalis*, *H. influenzae* (without producing beta-lactamases), *S. pyogenes*, *P. mirabilis*, *MRSA*	(Ewig et al. 2021; PEG 2019; AWMF 2024a)
2	Cefuroxime axetil	*P. mirabilis*	3.6%	*S. pneumoniae*, *H. influenzae*,* MSSA*	(PEG 2019; AWMF 2024a)
3	Doxycycline	*-*	-	*M. pneumoniae*, *C. trachomatis*, *Rickettsia* spp., *B. burgdorferi*, *M. pneumoniae*, *T. pallidum*,* F. tularensis*	(AWMF 2016; AWMF 2018; AWMF 2019b)
4	Amoxicillin clavulanic acid	*E. faecium*, ***E. faecalis***, ***S. pneumoniae***	77.5%; 0.0%; 0.9%	*H. influenzae*, *M. catarrhalis*, *S. pneumoniae*, *Prevotella* spp., *Porphyromonas* spp., *E. coli*, *P. mirabilis*, *E. faecalis*, *B. fragilis*, *P. multocida*, *Fusobacterium* spp.,* MSSA*	(PEG 2019; AWMF 2024a; AWMF 2019a)
5	Clindamycin	*-*	-	*Anaerobia*, *S. pyogenes*, *S. aureus*, *A. israelii*,* C. perfringens*	(PEG 2019; AWMF 2024a; AWMF 2020; AWMF 2024b)
6	Azithromycin	*-*	-	*M. pneumoniae, C. trachomatis, Campylobacter spp., L. pneumophila, S. pneumoniae, H. influenzae, M. catarrhalis; N. gonorrhoeae, B. pertussis, C. jejuni*	(Ewig et al. 2021; AWMF 2020; AWMF 2024b)
7	Sulfamethoxazole-trimethoprim	*P. aeruginosa*, *S. epidermidis*	4.9%; 16.6%	*S. maltophilia*, *P. jiroveci*, *E. coli*, *S. maltophilia*, *Nocardia* spp., *Toxoplasmose*, *H. influenzae*, *Shigella* spp., *Salmonella* spp., *L. monocytogenes*	(AWMF 2024a; AWMF 2020)
8	Nitrofurantoin	***E. coli***	0.9%	*E. coli*, *E. faecalis*,* S. saprophyticus*	(AWMF 2024a; PEG 2019)
9	Ciprofloxacin	*A. baumanii*, *C. freundii*, *C. oxytoca*, *E. cloacae*, ***K. pneumoniae***, *M. morganii*, *S. marcescens*	3.3%; 4.9%; 2.2%; 2.9%; 8.8%; 11.0%; 5.7%	*E. coli*, *K. pneumoniae*, *P. mirabilis*, *P. aeruginosa*, *Klebsiella* spp., *Enterobacter* spp., *Proteus* spp., *Salmonella* spp.*, Shigella* spp., *N. gonorrhoeae*, *B. anthracis*, *H. influenzae*, *M. catarrhalis*,* C. jejuni*	(PEG 2019; AWMF 2024a; AWMF 2020)
10	Clarithromycin	*-*	-	*M. pneumoniae*, *C. pneumoniae*, *L. pneumophila*, *S. pneumoniae*, *H. influenzae*, *M. catarrhalis*, *H. pylori*, *M. avium complex*	(PEG 2019; AWMF 2020; AWMF 2024a)

Actual AWMF and PEG guidelines can be accessed online (https://www.awmf.org/leitlinien; https://www.p-e-g.org/leitlinienempfehlungen.html). Besides the findings of our analysis for the TOP10, the assessment of German guidelines is given. If both recommendations agree, the antibacterial drug is shown in bold, if not, it is underlined. In general, the guidelines mention that an antibacterial drug that is not the first choice, however, can be used for treatment if the susceptibility of the respective pathogen is ensured
7. **The long-term perspective:** If points 1–6 are followed consistently, in the long run, bacterial resistance will decrease, bacterial infections will be treated more effectively, and costs for health care systems will be reduced.
8. **Get out of the “global antibiotic crisis” by applying the principles of science:** The pressure to develop new (and expensive) antibacterial drugs will be reduced. There is no “global antibiotic crisis” if the above-mentioned simple scientific rules are followed dilligently.
9. **Update therapeutic guidelines more frequently:** Therapeutic guidelines are often considered the “holy grail” for pharmacotherapy. In the area of bacterial infections, however, frequent updates are neede to ensure optimal drug therapy.

## Supplementary Information

Below is the link to the electronic supplementary material.

Supplementary file1 (DOCX 2855 KB)

## Data Availability

All source data for this study are available upon reasonable request from the authors.

## Abbreviations

AVR: Arzneiverordnungsreport (Drug Prescription Report regarding the GKV system of Germany)
AMR: Antimicrobial resistance
ARS: Antibiotika-Resistenz-Surveillance (surveillance of the development of bacterial resistance in antibacterial drugs)
DDD: Defined daily dose
GKV: gesetzliche Krankenversicherung (statutory health insurance)
RKI: Robert Koch Institute
SPSS: Statistical Product and Service Solutions (software for statistical analyses, IBM)
TOP15: Group of the 15 most prescribed antibacterial drugs (based on the year 2022)
TOP10: Group of the ten most prescribed antibacterial drugs (based on the year 2022)
TOP5: Group of the five most prescribed antibacterial drugs (based on the year 2022)
