# Supplementary material for: Development of bacterial resistance in Germany from 2008 to 2022 — major culprit pathogens, antibacterial drugs, and prescribing practices
Source: Naunyn Schmiedebergs Arch Pharmacol. 2024 Oct 23;398(4):4219–36. doi: 10.1007/s00210-024-03533-6 (PMC11978701; doi:10.1007/s00210-024-03533-6)

**Development of bacterial resistance in Germany from 2008 to 2022–**

**major culprit pathogens, antibacterial drugs and prescribing practices**

**Lilly Josephine Bindel and Roland Seifert**

Supplemental Results

**Amoxicillin: development of bacterial resistance and correlations between pathogens**

Amoxicillin belongs to the substance group of aminopenicillins and is the most prescribed antibacterial drug in 2022 (Ludwig et al. 2024). The bacterial resistance data was provided for five pathogens: *E. coli*, *E. faecalis, E. faecium, P. mirabilis* and *S. pneumoniae*. Data can be found in Table S1 as well as a graphical visualisation in Fig. S1.

When examining the development of bacterial resistance for analysed pathogens, there are rising as well as declining bacterial resistances, although there is a dominance of rising bacterial resistance (see Fig. S1). Three pathogens are rising, including *S. pneumoniae*, *P. mirabilis* and *E. faecium*. Only for *E. coli,* a declining bacterial resistance is depicted. *E. faecalis* remained stable in comparison of the first available (2020) till the latest (2022) data. With regard to significant correlations between the pathogens, there is a dominance of rising bacterial resistance. While *E. faecium* and *P. mirabilis* are rising, *E. coli* is declining.

Significant correlations among pathogens included a strong negative correlation between *E. coli* and *E. faecium* (**-1.000**) and a positive correlation between *E. coli* and *P. mirabilis* (**0.724**). The three pathogens *E. coli, E. faecium* and *P. mirabilis* have at least one significant correlation, while the two pathogens *E. faecalis* and *S. pneumoniae* exhibit only non-significant correlations.

**Cefuroxime axetil: development of bacterial resistance and correlations between pathogens**

Cefuroxime axetil belongs to the substance group of cephalosporins and is the second most prescribed antibacterial drug in 2022 (Ludwig et al. 2024). Resistance data included seven pathogens: *E. coli, C. oxytoca, K. pneumoniae, P. mirabilis, S. aureus, S. epidermidis* and *S. pneumoniae*. Data can be found in Table S2 as well as a graphical visualisation in Fig. S2.

When examining the development of bacterial resistance for analysed pathogens, nearly all pathogens show a rise in its bacterial resistance, including *C. oxytoca, E. coli, P. mirabilis, S. epidermidis, K. pneumoniae* and *S. pneumoniae* (see Fig. S2). *S. aureus* is the only pathogen that is declining during the examined period.

Between the pathogens, significant, strong and non-significant correlations are depicted. Nevertheless, all analysed pathogens have at least one significant correlation with another pathogen. *E. coli* exhibits three significantly positive and two negative correlations with *K. pneumoniae* (**0.962**), *P. mirabilis* (**0.726**), *S. pneumoniae* (**0.561**), *C. oxytoca* (**-0.595**) and *S. aureus* (**-0.777**). *C. oxytoca* is positively correlated with *S. aureus* (**0.674**) and *S. epidermidis* (**0.577**). *K. pneumoniae* exhibits positive correlations with *P. mirabilis* (**0.794**) and *S. pneumoniae* (**0.588**) and a negative correlation with *S. aureus* (**0.674**). *P. mirabilis* has a positive correlation with *S. pneumoniae* (**0.842**) and a negative correlation with *S. aureus* (**-0.799**). *S. pneumoniae* is negatively correlated with *S. aureus* (**-0.846**). Correlations considered as strong are depicted for *E. coli* with *K. pneumoniae* (**0.962**), as well as for *S. pneumoniae* with *P. mirabilis* (**0.842**) and *S. aureus* (**-0.846**).

**Doxycycline: development of bacterial resistance and correlations between pathogens**

Doxycycline, a tetracycline, was the third most prescribed antibacterial drug in 2022 (Ludwig et al. 2024). The bacterial resistance data was provided for the three pathogens *S. aureus, S. epidermidis* and *S. pneumoniae*. Data can be found in Table S3 as well as a graphical visualisation in Fig. S3.

Bacterial resistance is declining for the two pathogens *S. aureus* and *S. pneumoniae*, but rising in *S. epidermidis* (see Fig. S3). Nevertheless, no development of bacterial resistance is significantly correlated between analysed pathogens.

No significant correlations were found among the pathogens.

**Amoxicillin clavulanic acid: development of bacterial resistance and correlations between pathogens**

Amoxicillin clavulanic acid, another aminopenicillin, ranked fourth in prescriptions in 2022 (Ludwig et al. 2024). Resistance data included nine pathogens: *E. coli*, *E. faecalis*, *E. faecium*, *C. oxytoca*, *K. pneumoniae*, *P. mirabilis*, *S. aureus*, *S. epidermidis* and *S. pneumoniae*. Data can be found in Table S4 as well as a graphical visualisation in Fig. S4.

Six of nine pathogens show a rising bacterial resistance during the examined period, including *E. coli, E. faecalis, E. faecium, K. pneumoniae, P. mirabilis* and *S. pneumoniae.* Only the two pathogens *S. epidermidis* and *C. oxytoca* show a decline in its bacterial resistance (see Fig. S4). With regard to significant correlations between pathogens, all pathogens except *S. aureus* and *S. epidermidis* are rising, including *E. faecium, E. coli, K. pneumoniae, P. mirabilis* and *S. pneumoniae.*

Between the pathogens, many correlations are significant, which are both negative and positive. Significant correlations are depicted for *E. faecium, E. coli, K. pneumoniae, P. mirabilis, S. aureus, S. pneumoniae* and *S. epidermidis*. Only *E. faecalis* and *C. oxytoca* have no significant correlations. The majority of the significant correlations are strong, with a correlation coefficient exceeding 0.8. Strong positive correlations are depicted for *E. coli* with *K. pneumoniae* (**0.932**) and *P. mirabilis* (**0.973**), *K. pneumoniae* with *P. mirabilis* (**0.896**) and *S. epidermidis* with *S. aureus* (**0.820**). A strong negative correlation exists between *S. aureus* with *S. pneumoniae* (**-0.800**). A significant, but not strong correlation was observed between *S. epidermidis* and *S. pneumoniae* (**-0.524**).

**Clindamycin: development of bacterial resistance and correlations between pathogens**

Clindamycin is a lincosamide and is the fifth most prescribed antibacterial drug in 2022 (Ludwig et al. 2024). The bacterial resistance data includes results for the three pathogens *S. aureus, S. epidermidis* and *S. pneumoniae*. Data can be found in Table S5 as well as a graphical visualisation in Fig. S5.

With regard to the development of bacterial resistance, all three examined pathogens show a declining trend, including the two significant pathogens *S. aureus* and *S. epidermidis* (see Fig. S5).

One significant positive correlation is depicted for bacterial resistance between pathogens, which is considered as strong since its correlation parameter exceeds (+/-) 0.8. This correlation is exhibited for *S. aureus* with *S. epidermidis* (**0.866**). All other correlations are non-significant.

**Azithromycin: development of bacterial resistance and correlations between pathogens**

Azithromycin belongs to the macrolides and is the sixth most prescribed antibacterial drug in 2022 (Ludwig et al. 2024). The bacterial resistance data includes results for *S. aureus. S. epidermidis* and *S. pneumoniae*. Data can be found in Table S6 as well as a graphical viusualisation in Fig. S6.

A declining trend of bacterial resistance is depicted for all three pathogens *S. aureus, S. epidermidis* and *S. pneumoniae*, although none of them is significantly correlated with each other.

There are no significant correlations between the pathogens.

**Sulfamethoxazole-Trimethoprim: development of bacterial resistance and correlations between pathogens**

Sulfamethoxazole-trimethoprim belongs to the substance group of sulfonamides and is the seventh most prescribed antibacterial drug in 2022 (Ludwig et al. 2024). The bacterial resistance data was available for the nine pathogens *E. coli, C. freundii, E. cloacae, C. oxytoca, K. pneumoniae, M. morganii, P. aeruginosa, S. aureus* and *S. epidermidis*. Data can be found in Table S7 as well as a graphical visualisation in Fig. S7.

Six of nine pathogens show a rising bacterial resistance during the examined period, including *C. freundii, E. cloacae, P. aeruginosa, S. aureus, K. pneumoniae* and *S. epidermidis.* A decline in bacterial resistance is depicted for the two pathogens *E. coli* and *M. morganii*. For *C. oxytoca*, the values of bacterial resistance remained stable when comparing the first (2011) with the last data (2022). With regard to significant correlations between pathogens, there are five rising, two declining and one stable pathogen, since the rising *C. freundii* is considered as non-significant.

Various significant correlations between the pathogens are recognisable. *C. freundii* is the only pathogen that does not have any significant correlations. *S. epidermidis* has positive correlations with nearly all other pathogens. Exceptions are the strong negative correlation with *S. aureus* (**-0.860**) and a non-significant correlation with *C. freundii. S. aureus* shows a strong negative correlation with *E. coli* (**-0.882**), *C. oxytoca* (**-0.819**) and a significant but not strong correlation with *M. morganii* (**-0.795**). *P. aeruginosa* has a positive correlation with *M. morganii* (**0.728**) and *K. pneumoniae* (**0.603**). *M. morganii* is positively correlated with *C. oxytoca* (**0.724**), *E. cloacae* (**0.616**) and *K. pneumoniae* (**0.582**). *K. pneumoniae* shows a strong positive significance with *E. cloacae* (**0.838**) and a significant but not strong correlation with *C. oxytoca* (**0.600**).

Strong correlations are both positive and negative. Strong positive correlations are exhibited for *S. epidermidis* with *E. coli* (**0.993**), *E. cloacae* (**0.898**), *C. oxytoca* (**0.974**) and *M. morganii* (**0.981**), as well as for *K. pneumoniae* with *E. cloacae* (**0.838**). Strong negative correlations are depicted for *S. aureus* with *E. coli* (**-0.882**), *C. oxytoca* (**-0.819**) and *S. epidermidis* (**-0.860**).

**Nitrofurantoin: development of bacterial resistance and correlations between pathogens**

Nitrofurantoin belongs to the group of “other anti-infective chemotherapeutics” and is the ninth most prescribed antibacterial drug in 2022 (Ludwig et al. 2024). Since there was no data provided for phenoxymethylpenicillin, which originally ranked before nitrofurantoin, it became ranked as number 8. The bacterial resistance data includes results for *E. coli, E. faecalis, P. mirabilis, S. aureus* and *S. epidermidis*. Data can be found in Table S8 as well as a graphical visualisation in Fig. S8.

When examining the development of bacterial resistance for analysed pathogens, nearly all pathogens show a rise in its bacterial resistance, including *E. faecalis, P. mirabilis, S. aureus* and *S. epidermidis*. *E. coli* is the only declining pathogen within the examined period. When considering only significant correlations, the three pathogens *P. mirabilis, S. aureus* and *S. epidermidis* are rising while only *E. coli* shows a declining bacterial resistance.

Significant, strong and non-significant correlations are available within the correlations between pathogens. Except *E. faecalis*, all pathogens have several significant correlations within each other. Significant and strong correlations are both positive and negative. One strong positive and one strong negative correlation is depicted for *S. epidermidis* with *S. aureus* (**0.958**) and *E. coli* (**-0.804**). Another strong negative correlation exists between *S. aureus* and *E. coli* (**-0.830**). Significant, but not strong correlations are shown for *P. mirabilis* (**-0.693**) with *E. coli* (**-0.693**), *S. aureus* (**0.582**) and *S. epidermidis* (**0.656**).

**Ciprofloxacin: development of bacterial resistance and correlations between pathogens**

Ciprofloxacin is a fluoroquinolone and the tenth most prescribed antibacterial drug in 2022 (Ludwig et al. 2024), but ranked as number 9 here. The bacterial resistance data included *E. coli, A. baumanii, C. freundii, E. cloacae, E. faecalis, E. faecium, C. oxytoca, K. pneumoniae, M. morganii, P. aeruginosa, S. marcescens, S. aureus* and *S. epidermidis*. Data can be found in Table S9 as well as a graphical visualisation in Fig. S9.

With regard to the development of bacterial resistance, nine pathogens show a declining trend during the examined period. In contrast, a rise is depicted for the five pathogens *E. faecium, E. faecalis, K. pneumoniae, M. morganii* and *P. mirabilis*. With regard to significant correlations, 8 of 12 pathogens depict a declining trend, including *E. coli*, *A. baumanii, E. cloacae, C. oxytoca, P. aeruginosa, S. marcescens, S. aureus* and *S. epidermidis.* The other four significant pathogens are rising, including *E. faecium, K. pneumoniae, M. morganii* and *P. mirabilis.*

Between the pathogens, various significant correlations are exhibited, which are be both positive and negative. *E. coli* has only significantly positive correlations, which are depicted with *A. baumanii* (**0.690**), *E. cloacae* (**0.819**), *C. oxytoca* (**0.809**), *P. aeruginosa* (**0.625**), *S. aureus* (**0.858**) and *S. epidermidis* (**0.712**). *A. baumanii* exhibits positive correlations with *E. cloacae* (**0.743**), *C. oxytoca* (**0.945**), *S. marcescens* (**0.514**) and *S. aureus* (**0.573**). *E. cloacae* is positively correlated with *C. oxytoca* (**0.784**) and *S. marcescens* (**0.609**), *E. faecalis* with *M. morganii* (**0.524**) and *P. mirabilis* (**0.645**), *K. pneumoniae* with *M. morganii* (**0.794**) and *P. mirabilis* (**0.750**), as well as *S. aureus* with *S. epidermidis* (**0.814**) and *C. oxytoca* (**0.721**). Significant negative correlations are depicted for *A. baumanii* with *K. pneumoniae* (**-0.546**), *M. morganii* (**-0.786**) and *P. mirabilis* (**-0.831**), as well as for *C. oxytoca* with *M. morganii* (**-0.691**) and *P. mirabilis* (**-0.706**). Only non-significant correlations are depicted for *E. faecium* and *C. freundii*.

Strong positive correlations, defined with a correlation coefficient exceeding (+/-) 0.8, are exhibited for the pathogens *E. coli* with *E. cloacae* (**0.819**) and *C. oxytoca* (**0.809**), *C. oxytoca* with *A. baumanii* (**0.945**), *M. morganii* with *P. mirabilis* (**0.944**), as well as for *S. epidermidis* with *S. aureus* (**0.814**). A strong negative correlation exists between *P. mirabilis* and *A. baumanii* (**-0.831**).

**Clarithromycin: development of bacterial resistance and correlations between pathogens**

Clarithromycin belongs to the substance group of macrolides and is the eleventh most prescribed antibacterial drug in 2022 (Ludwig et al. 2024), but ranked as no. 10 here. The bacterial resistance data includes results for *S. aureus*, S. *epidermidis* and *S. pneumoniae*. Data can be found in Table S10 as well as a graphical visualisation in Fig. S10.

All three pathogens have a declining trend of bacterial resistance during the examined period.

There are no significant correlations between the bacterial resistance developments of the pathogens.

**Interactions between pathogens – Analysis of significant correlations**

| The total number of significant correlations offers an initial indication of the concurrent development of bacterial resistance. A higher number of significant correlations suggests that pathogens or drugs exhibit similar resistance patterns, while a lower or near-zero value indicates a lack of similarities. Pathogens with a larger number of analysed drugs tend to have a greater number of significant correlations due to the higher number of possible comparisons with other pathogens. However, a large number of comparisons does not necessarily lead to a large number of significant correlations. An overview about the following results is depicted in Table 3 and 4.  For antibacterial drugs, the number of significant correlations ranges from zero for doxycycline, azithromycin and clarithromycin, to 25 for ciprofloxacin. Amoxicillin (**2**) and clindamycin (**1**) exhibit relatively low numbers of significant correlations, whereas sulfamethoxazole-trimethoprim (**17**) and cefuroxime axetil (**13**) show higher numbers. Nitrofurantoin (**6**) and amoxicillin clavulanic acid (**7**) fall in the intermediate range. The absence of significant correlations in some cases is attributable to the non-significance of the results. Ciprofloxacin exhibits the highest number of significant correlations, which could be partially explained by the large number of pathogens analyzed.  Regarding pathogens, the number of significant correlations varies widely, from zero for *C. freundii* to 21 for both *E. coli* and *S. aureus*. Lower counts are observed for *E. faecium* (**1**), *E. faecalis* (**2**), *S. marcescens* (**2**) and *P. aeruginosa* (**3**). In contrast, *S. epidermidis* (**17**), *P. mirabilis* (**16**), *K. pneumoniae* (**14**) and *C. oxytoca* (13) exhibit higher numbers.  Supplemental Tables | | | | | | |  |
| --- | --- | --- | --- | --- | --- | --- | --- |
| ***Table S1:*** *Correlation matrix, generated by SPSS, of the correlations between the bacterial resistance of analysed pathogens for amoxicillin from 2008-2022. Dark green colour and “**” indicates a significant correlation at the 0.01 level. Light green colour and “*” indicates a significant correlation at the 0.05 level. Orange colour indicates no significant correlation.* | | | | | | |  |
| 1 Amoxicillin | | *E. coli* | *E. faecalis* bacterial resistance | *E. faecium* bacterial resistance | *P. mirabilis* bacterial resistance | *S. pneumoniae* bacterial resistance | |
|  |  | bacterial resistance |  |  |  |  |  |
| *E. coli* | Pearson Correlation | -- |  |  |  |  | |
| bacterial resistance | Sig. (2-tailed) |  |  |  |  |  | |
|  | R^2^ |  |  |  |  |  | |
|  | N | 15 |  |  |  |  | |
| *E. faecalis* | Pearson Correlation | -0.352 | -- |  |  |  | |
| bacterial resistance | Sig. (2-tailed) | 0.392 |  |  |  |  | |
|  | R^2^ | 0.124 |  |  |  |  | |
|  | N | 8 | 8 |  |  |  | |
| *E. faecium* | Pearson Correlation | -1.000^*^ | 0.017 | -- |  |  | |
| bacterial resistance | Sig. (2-tailed) | 0.011 | 0.989 |  |  |  | |
|  | R^2^ | 1 | 0 |  |  |  | |
|  | N | 3 | 3 | 3 |  |  | |
| *P. mirabilis* | Pearson Correlation | 0.724^**^ | -0.265 | -0.875 | -- |  | |
| bacterial resistance | Sig. (2-tailed) | 0.002 | 0.527 | 0.322 |  |  | |
|  | R^2^ | 0.524 | 0.07 | 0.766 |  |  | |
|  | N | 15 | 8 | 3 | 15 |  | |
| *S. pneumoniae* | Pearson Correlation | -0.491 | -0.031 | -0.413 | -0.036 | -- | |
| bacterial resistance | Sig. (2-tailed) | 0.063 | 0.943 | 0.729 | 0.898 |  | |
|  | R^2^ | 0.241 | 0.001 | 0.171 | 0.001 |  | |
|  | N | 15 | 8 | 3 | 15 | 15 | |

***Table S2:*** *Correlation matrix, generated by SPSS, of the correlations between the bacterial resistance of analysed pathogens for cefuroxime axetil from 2008-2022. Dark green colour and “**” indicates a significant correlation at the 0.01 level. Light green colour and “*” indicates a significant correlation at the 0.05 level. Orange colour indicates no significant correlation.*

| 2 Cefuroxime axetil | | *E. coli* bacterial resistance | *C. oxytoca* bacterial resistance | *K. pneumoniae* bacterial resistance | *P. mirabilis* bacterial resistance | *S. aureus* bacterial resistance | *S. epidermidis* bacterial resistance | *S. pneumoniae* bacterial resistance |
| --- | --- | --- | --- | --- | --- | --- | --- | --- |
| *E. coli* | Pearson Correlation | -- |  |  |  |  |  |  |
| bacterial resistance | Sig. (2-tailed) |  |  |  |  |  |  |  |
|  | R^2^ |  |  |  |  |  |  |  |
|  | N | 15 |  |  |  |  |  |  |
| *C. oxytoca* | Pearson Correlation | -0.595^*^ | -- |  |  |  |  |  |
| bacterial resistance | Sig. (2-tailed) | 0.019 |  |  |  |  |  |  |
|  | R^2^ | 0.354 |  |  |  |  |  |  |
|  | N | 15 | 15 |  |  |  |  |  |
| *K. pneumoniae* | Pearson Correlation | 0.962^**^ | -0.427 | -- |  |  |  |  |
| bacterial resistance | Sig. (2-tailed) | 0 | 0.113 |  |  |  |  |  |
|  | R^2^ | 0.925 | 0.182 |  |  |  |  |  |
|  | N | 15 | 15 | 15 |  |  |  |  |
| *P. mirabilis* | Pearson Correlation | 0.726^**^ | -0.382 | 0.794^**^ | -- |  |  |  |
| bacterial resistance | Sig. (2-tailed) | 0.002 | 0.16 | 0 |  |  |  |  |
|  | R^2^ | 0.527 | 0.146 | 0.63 |  |  |  |  |
|  | N | 15 | 15 | 15 | 15 |  |  |  |
| *S. aureus* | Pearson Correlation | -0.777^**^ | 0.674^**^ | -0.734^**^ | -0.799^**^ | -- |  |  |
| bacterial resistance | Sig. (2-tailed) | 0.001 | 0.006 | 0.002 | 0 |  |  |  |
|  | R^2^ | 0.604 | 0.454 | 0.539 | 0.638 |  |  |  |
|  | N | 15 | 15 | 15 | 15 | 15 |  |  |
| *S. epidermidis* | Pearson Correlation | 0.071 | 0.577^*^ | 0.206 | -0.011 | 0.234 | -- |  |
| bacterial resistance | Sig. (2-tailed) | 0.802 | 0.024 | 0.462 | 0.97 | 0.401 |  |  |
|  | R^2^ | 0.005 | 0.333 | 0.424 | 0 | 0.055 |  |  |
|  | N | 15 | 15 | 15 | 15 | 15 | 15 |  |
| *S. pneumoniae* | Pearson Correlation | 0.561^*^ | -0.371 | 0.588^*^ | 0.842^**^ | -0.846^**^ | -0.059 | -- |
| bacterial resistance | Sig. (2-tailed) | 0.03 | 0.174 | 0.021 | 0 | 0 | 0.835 |  |
|  | R^2^ | 0.315 | 0.138 | 0.346 | 0.709 | 0.716 | 0.003 |  |
|  | N | 15 | 15 | 15 | 15 | 15 | 15 | 15 |

***Table S3:*** *Correlation matrix, generated by SPSS, of the correlations between the bacterial resistance of analysed pathogens for doxycycline from 2008-2022. Dark green colour and “**” indicates a significant correlation at the 0.01 level. Light green colour and “*” indicates a significant correlation at the 0.05 level. Orange colour indicates no significant correlation.*

| 3 Doxycycline | | *S. aureus*  bacterial resistance | *S. epidermidis*  bacterial resistance | *S. pneumoniae*  bacterial resistance |
| --- | --- | --- | --- | --- |
| *S. aureus* | Pearson Correlation | -- |  |  |
| bacterial resistance | Sig. (2-tailed) |  |  |  |
|  | R^2^ |  |  |  |
|  | N | 15 |  |  |
| *S. epidermidis* | Pearson Correlation | 0.335 | -- |  |
| bacterial resistance | Sig. (2-tailed) | 0.222 |  |  |
|  | R^2^ | 0.112 |  |  |
|  | N | 15 | 15 |  |
| *S. pneumoniae* | Pearson Correlation | 0.386 | -0.418 | -- |
| bacterial resistance | Sig. (2-tailed) | 0.156 | 0.121 |  |
|  | R^2^ | 0.149 | 0.175 |  |
|  | N | 15 | 15 | 15 |

***Table S4:*** *Correlation matrix, generated by SPSS, of the correlations between the bacterial resistance of analysed pathogens for amoxicillin clavulanic acid from 2008-2022. Dark green colour and “**” indicates a significant correlation at the 0.01 level. Light green colour and “*” indicates a significant correlation at the 0.05 level. Orange colour indicates no significant correlation.*

| 4 Amoxicillin  clavulanic acid | | *E. coli* bacterial resistance | *E. faecalis* bacterial resistance | *E. faecium* bacterial resistance | *C. oxytoca* bacterial resistance | *K. pneumoniae* bacterial resistance | *P. mirabilis* bacterial resistance | *S. aureus* bacterial resistance | *S. epidermidis* bacterial resistance | *S. pneumoniae* bacterial resistance |
| --- | --- | --- | --- | --- | --- | --- | --- | --- | --- | --- |
| *E. coli* | Pearson Correlation | -- |  |  |  |  |  |  |  |  |
| bacterial resistance | Sig. (2-tailed) |  |  |  |  |  |  |  |  |  |
|  | R^2^ |  |  |  |  |  |  |  |  |  |
|  | N | 15 |  |  |  |  |  |  |  |  |
| *E. faecalis* | Pearson Correlation | -0.08 | -- |  |  |  |  |  |  |  |
| bacterial resistance | Sig. (2-tailed) | 0.826 |  |  |  |  |  |  |  |  |
|  | R^2^ | 0.006 |  |  |  |  |  |  |  |  |
|  | N | 10 | 10 |  |  |  |  |  |  |  |
| *E. faecium* | Pearson Correlation | -0.955^*^ | -0.042 | -- |  |  |  |  |  |  |
| bacterial resistance | Sig. (2-tailed) | 0.045 | 0.958 |  |  |  |  |  |  |  |
|  | R^2^ | 0.912 | 0.002 |  |  |  |  |  |  |  |
|  | N | 4 | 4 | 4 |  |  |  |  |  |  |
| *C. oxytoca* | Pearson Correlation | 0.44 | -0.322 | -0.866 | -- |  |  |  |  |  |
| bacterial resistance | Sig. (2-tailed) | 0.101 | 0.364 | 0.134 |  |  |  |  |  |  |
|  | R^2^ | 0.194 | 0.104 | 0.75 |  |  |  |  |  |  |
|  | N | 15 | 10 | 4 | 15 |  |  |  |  |  |
| *K. pneumoniae* bacterial resistance | Pearson Correlation | 0.932^**^ | -0.248 | -0.469 | 0.382 | -- |  |  |  |  |
|  | Sig. (2-tailed) | 0 | 0.489 | 0.531 | 0.16 |  |  |  |  |  |
|  | R^2^ | 0.869 | 0.062 | 0.22 | 0.146 |  |  |  |  |  |
|  | N | 15 | 10 | 4 | 15 | 15 |  |  |  |  |
| *P. mirabilis* | Pearson Correlation | 0.973^**^ | -0.183 | -0.688 | 0.349 | 0.896^**^ | -- |  |  |  |
| bacterial resistance | Sig. (2-tailed) | 0 | 0.612 | 0.312 | 0.202 | 0 |  |  |  |  |
|  | R^2^ | 0.947 | 0.335 | 0.473 | 0.122 | 0.534 |  |  |  |  |
|  | N | 15 | 10 | 4 | 15 | 15 | 15 |  |  |  |
| *S. aureus* | Pearson Correlation | -0.443 | -0.438 | -0.867 | 0.468 | -0.475 | -0.508 | -- |  |  |
| bacterial resistance | Sig. (2-tailed) | 0.098 | 0.206 | 0.133 | 0.079 | 0.074 | 0.053 |  |  |  |
|  | R^2^ | 0.196 | 0.192 | 0.751 | 0.219 | 0.226 | 0.258 |  |  |  |
|  | N | 15 | 10 | 4 | 15 | 15 | 15 | 15 |  |  |
| *S. epidermidis* | Pearson Correlation | -0.254 | -0.401 | 0.088 | 0.422 | -0.233 | -0.344 | 0.820^**^ | -- |  |
| bacterial resistance | Sig. (2-tailed) | 0.362 | 0.251 | 0.912 | 0.117 | 0.403 | 0.21 | 0 |  |  |
|  | R^2^ | 0.064 | 0.161 | 0.008 | 0.178 | 0.054 | 0.118 | 0.672 |  |  |
|  | N | 15 | 10 | 4 | 15 | 15 | 15 | 15 | 15 |  |
| *S. pneumoniae* bacterial resistance | Pearson Correlation | 0.359 | 0.115 | 0.675 | -0.449 | 0.404 | 0.403 | -0.800^**^ | -0.524^*^ | -- |
|  | Sig. (2-tailed) | 0.188 | 0.751 | 0.325 | 0.093 | 0.136 | 0.137 | 0 | 0.045 |  |
|  | R^2^ | 0.129 | 0.013 | 0.456 | 0.202 | 0.163 | 0.162 | 0.64 | 0.275 |  |
|  | N | 15 | 10 | 4 | 15 | 15 | 15 | 15 | 15 | 15 |

***Table S5:*** *Correlation matrix, generated by SPSS, of the correlations between the bacterial resistance of analysed pathogens for clindamycin from 2008-2022. Dark green colour and “**” indicates a significant correlation at the 0.01 level. Light green colour and “*” indicates a significant correlation at the 0.05 level. Orange colour indicates no significant correlation.*

| 5 Clindamycin | | *S. aureus* | *S. epidermidis* bacterial resistance | *S. pneumoniae* bacterial resistance |
| --- | --- | --- | --- | --- |
|  |  | bacterial resistance |  |  |
| *S. aureus* | Pearson Correlation | -- |  |  |
| bacterial resistance | Sig. (2-tailed) |  |  |  |
|  | R^2^ |  |  |  |
|  | N | 15 |  |  |
| *S. epidermidis* | Pearson Correlation | 0.866^**^ | -- |  |
| bacterial resistance | Sig. (2-tailed) | 0 |  |  |
|  | R^2^ | 0.75 |  |  |
|  | N | 15 | 15 |  |
| *S. pneumoniae* | Pearson Correlation | -0.058 | 0.223 | -- |
| bacterial resistance | Sig. (2-tailed) | 0.839 | 0.423 |  |
|  | R^2^ | 0.003 | 0.497 |  |
|  | N | 15 | 15 | 15 |

***Table S6:*** *Correlation matrix, generated by SPSS, of the correlations between the bacterial resistance of analysed pathogens for azithromycin from 2008-2022. Dark green colour and “**” indicates a significant correlation at the 0.01 level. Light green colour and “*” indicates a significant correlation at the 0.05 level. Orange colour indicates no significant correlation.*

| 6 Azithromycin | | *S. aureus* | *S. epidermidis* bacterial resistance | *S. pneumoniae* bacterial resistance |
| --- | --- | --- | --- | --- |
|  |  | bacterial resistance |  |  |
| *S. aureus* | Pearson Correlation | -- |  |  |
| bacterial resistance | Sig. (2-tailed) |  |  |  |
|  | R^2^ |  |  |  |
|  | N | 15 |  |  |
| *S. epidermidis* | Pearson Correlation | 0.415 | -- |  |
| bacterial resistance | Sig. (2-tailed) | 0.124 |  |  |
|  | R^2^ | 0.172 |  |  |
|  | N | 15 | 15 |  |
| *S. pneumoniae* | Pearson Correlation | -0.657 | -0.028 | -- |
| bacterial resistance | Sig. (2-tailed) | 0.054 | 0.943 |  |
|  | R^2^ | 0.431 | 0.001 |  |
|  | N | 9 | 9 | 9 |

***Table S7:*** *Correlation matrix, generated by SPSS, of the correlations between the bacterial resistance of analysed pathogens for sulfamethoxazole-trimethoprim from 2008-2022. Dark green colour and “**” indicates a significant correlation at the 0.01 level. Light green colour and “*” indicates a significant correlation at the 0.05 level. Orange colour indicates no significant correlation.*

| 7 Sulfamethoxazole-Trimethoprim | | *E. coli*  bacterial resistance | *C. freundii*  bacterial resistance | *E. cloacae*  bacterial resistance | *C. oxytoca*  bacterial resistance | *K. pneumoniae* bacterial resistance | *M. morganii*  bacterial resistance | *P. aeruginosa*  bacterial resistance | *S. aureus*  bacterial resistance | *S. epidermidis*  bacterial resistance |
| --- | --- | --- | --- | --- | --- | --- | --- | --- | --- | --- |
| *E. coli* | Pearson Correlation | -- |  |  |  |  |  |  |  |  |
| bacterial resistance | Sig. (2-tailed) |  |  |  |  |  |  |  |  |  |
|  | R^2^ |  |  |  |  |  |  |  |  |  |
|  | N | 15 |  |  |  |  |  |  |  |  |
| *C. freundii* bacterial resistance | Pearson Correlation | 0.067 | -- |  |  |  |  |  |  |  |
|  | Sig. (2-tailed) | 0.835 |  |  |  |  |  |  |  |  |
|  | R^2^ | 0.004 |  |  |  |  |  |  |  |  |
|  | N | 12 | 12 |  |  |  |  |  |  |  |
| *E. cloacae* bacterial resistance | Pearson Correlation | -0.519 | 0.299 | -- |  |  |  |  |  |  |
|  | Sig. (2-tailed) | 0.084 | 0.345 |  |  |  |  |  |  |  |
|  | R^2^ | 0.269 | 0.089 |  |  |  |  |  |  |  |
|  | N | 12 | 12 | 12 |  |  |  |  |  |  |
| *C. oxytoca* bacterial resistance | Pearson Correlation | 0.402 | 0.329 | 0.48 | -- |  |  |  |  |  |
|  | Sig. (2-tailed) | 0.195 | 0.297 | 0.114 |  |  |  |  |  |  |
|  | R^2^ | 0.161 | 0.088 | 0.23 |  |  |  |  |  |  |
|  | N | 12 | 12 | 12 | 12 |  |  |  |  |  |
| *K. pneumoniae* bacterial resistance | Pearson Correlation | -0.339 | 0.383 | 0.838^**^ | 0.600^*^ | -- |  |  |  |  |
|  | Sig. (2-tailed) | 0.257 | 0.219 | 0.001 | 0.039 |  |  |  |  |  |
|  | R^2^ | 0.114 | 0.147 | 0.702 | 0.36 |  |  |  |  |  |
|  | N | 13 | 12 | 12 | 12 | 13 |  |  |  |  |
| *M. morganii* bacterial resistance | Pearson Correlation | 0.253 | 0.088 | 0.616^*^ | 0.724^**^ | 0.582^*^ | -- |  |  |  |
|  | Sig. (2-tailed) | 0.427 | 0.786 | 0.033 | 0.008 | 0.047 |  |  |  |  |
|  | R^2^ | 0.064 | 0.008 | 0.379 | 0.524 | 0.339 |  |  |  |  |
|  | N | 12 | 12 | 12 | 12 | 12 | 12 |  |  |  |
| *P. aeruginosa* bacterial resistance | Pearson Correlation | -0.167 | -0.295 | 0.566 | 0.292 | 0.603^*^ | 0.728^**^ | -- |  |  |
|  | Sig. (2-tailed) | 0.603 | 0.352 | 0.055 | 0.357 | 0.038 | 0.007 |  |  |  |
|  | R^2^ | 0.028 | 0.087 | 0.32 | 0.058 | 0.364 | 0.53 |  |  |  |
|  | N | 12 | 12 | 12 | 12 | 12 | 12 | 12 |  |  |
| *S. aureus* bacterial resistance | Pearson Correlation | -0.882^**^ | -0.516 | -0.694 | -0.819^*^ | -0.473 | -0.795^*^ | -0.346 | -- |  |
|  | Sig. (2-tailed) | 0.004 | 0.191 | 0.056 | 0.013 | 0.237 | 0.018 | 0.401 |  |  |
|  | R^2^ | 0.778 | 0.266 | 0.481 | 0.671 | 0.224 | 0.632 | 0.119 |  |  |
|  | N | 8 | 8 | 8 | 8 | 8 | 8 | 8 | 8 |  |
| *S. epidermidis* bacterial resistance | Pearson Correlation | 0.993^**^ | 0.409 | 0.898^**^ | 0.974^**^ | 0.759^*^ | 0.981^**^ | 0.708^*^ | -0.860^**^ | -- |
|  | Sig. (2-tailed) | 0 | 0.314 | 0.002 | 0 | 0.029 | 0 | 0.049 | 0.006 |  |
|  | R^2^ | 0.986 | 0.167 | 0.806 | 0.949 | 0.576 | 0.962 | 0.501 | 0.74 |  |
|  | N | 8 | 8 | 8 | 8 | 8 | 8 | 8 | 8 | 8 |

***Table S8:*** *Correlation matrix, generated by SPSS, of the correlations between the bacterial resistance of analysed pathogens for nitrofurantoin from 2008-2022. Dark green colour and “**” indicates a significant correlation at the 0.01 level. Light green colour and “*” indicates a significant correlation at the 0.05 level. Orange colour indicates no significant correlation.*

| 8 Nitrofurantoin | | *E. coli*  bacterial resistance | *E. faecalis* bacterial resistance | *P. mirabilis* bacterial resistance | *S. aureus* bacterial resistance | *S. epidermidis* bacterial resistance |
| --- | --- | --- | --- | --- | --- | --- |
| *E. coli* | Pearson Correlation | -- |  |  |  |  |
| bacterial resistance | Sig. (2-tailed) |  |  |  |  |  |
|  | R^2^ |  |  |  |  |  |
|  | N | 15 |  |  |  |  |
| *E. faecalis* | Pearson Correlation | -0.344 | -- |  |  |  |
| bacterial resistance | Sig. (2-tailed) | 0.364 |  |  |  |  |
|  | R^2^ | 0.118 |  |  |  |  |
|  | N | 9 | 9 |  |  |  |
| *P. mirabilis* | Pearson Correlation | -0.693^**^ | 0.401 | -- |  |  |
| bacterial resistance | Sig. (2-tailed) | 0.009 | 0.285 |  |  |  |
|  | R^2^ | 0.48 | 0.161 |  |  |  |
|  | N | 13 | 9 | 13 |  |  |
| *S. aureus* | Pearson Correlation | -0.830^**^ | 0.377 | 0.582^*^ | -- |  |
| bacterial resistance | Sig. (2-tailed) | 0 | 0.318 | 0.037 |  |  |
|  | R^2^ | 0.689 | 0.142 | 0.339 |  |  |
|  | N | 15 | 9 | 13 | 15 |  |
| *S. epidermidis* | Pearson Correlation | -0.804^**^ | 0.568 | 0.656^*^ | 0.958^**^ | -- |
| bacterial resistance | Sig. (2-tailed) | 0 | 0.111 | 0.015 | 0 |  |
|  | R^2^ | 0.646 | 0.323 | 0.43 | 0.918 |  |
|  | N | 15 | 9 | 13 | 15 | 15 |

***Table S9:*** *Correlation matrix, generated by SPSS, of the correlations between the bacterial resistance of analysed pathogens for ciprofloxacin from 2008-2022. Dark green colour and “**” indicates a significant correlation at the 0.01 level. Light green colour and “*” indicates a significant correlation at the 0.05 level. Orange colour indicates no significant correlation.*

| 9 Ciprofloxacin | | *E. coli* | *A. baumannii* bacterial resistance | *C. freundii* bacterial resistance | *E. cloacae* bacterial resistance | *E. faecalis* bacterial resistance | *E. faecium* bacterial resistance | *C. oxytoca* bacterial resistance | *K. pneumoniae* bacterial resistance | *M. morganii* bacterial resistance | *P. mirabilis* bacterial resistance | *P. aeruginosa* bacterial resistance | *S. marcescens* bacterial resistance | *S. aureus* bacterial resistance | *S. epidermidis* bacterial resistance |
| --- | --- | --- | --- | --- | --- | --- | --- | --- | --- | --- | --- | --- | --- | --- | --- |
|  |  | bacterial resistance |  |  |  |  |  |  |  |  |  |  |  |  |  |
| *E. coli* bacterial resistance | Pearson Correlation | -- |  |  |  |  |  |  |  |  |  |  |  |  |  |
|  | Sig. (2-tailed) |  |  |  |  |  |  |  |  |  |  |  |  |  |  |
|  | R^2^ |  |  |  |  |  |  |  |  |  |  |  |  |  |  |
|  | N | 15 |  |  |  |  |  |  |  |  |  |  |  |  |  |
| *A. baumannii* bacterial resistance | Pearson Correlation | 0.685^**^ | -- |  |  |  |  |  |  |  |  |  |  |  |  |
|  | Sig. (2-tailed) | 0.005 |  |  |  |  |  |  |  |  |  |  |  |  |  |
|  | R^2^ | 0.469 |  |  |  |  |  |  |  |  |  |  |  |  |  |
|  | N | 15 | 15 |  |  |  |  |  |  |  |  |  |  |  |  |
| *C. freundii* bacterial resistance | Pearson Correlation | 0.142 | 0.458 | -- |  |  |  |  |  |  |  |  |  |  |  |
|  | Sig. (2-tailed) | 0.613 | 0.086 |  |  |  |  |  |  |  |  |  |  |  |  |
|  | R^2^ | 0.02 | 0.209 |  |  |  |  |  |  |  |  |  |  |  |  |
|  | N | 15 | 15 | 15 |  |  |  |  |  |  |  |  |  |  |  |
| *E. cloacae* bacterial resistance | Pearson Correlation | 0.819^**^ | 0.743^**^ | 0.287 | -- |  |  |  |  |  |  |  |  |  |  |
|  | Sig. (2-tailed) | 0 | 0.002 | 0.299 |  |  |  |  |  |  |  |  |  |  |  |
|  | R^2^ | 0.67 | 0.552 | 0.082 |  |  |  |  |  |  |  |  |  |  |  |
|  | N | 15 | 15 | 15 | 15 |  |  |  |  |  |  |  |  |  |  |
| *E. faecalis* bacterial resistance | Pearson Correlation | 0.067 | -0.435 | -0.44 | -0.064 | -- |  |  |  |  |  |  |  |  |  |
|  | Sig. (2-tailed) | 0.813 | 0.105 | 0.1 | 0.821 |  |  |  |  |  |  |  |  |  |  |
|  | R^2^ | 0.004 | 0.189 | 0.194 | 0.004 |  |  |  |  |  |  |  |  |  |  |
|  | N | 15 | 15 | 15 | 15 | 15 |  |  |  |  |  |  |  |  |  |
| *E. faecium* bacterial resistance | Pearson Correlation | -0.615 | -0.597 | -0.197 | -0.597 | -0.513 | -- |  |  |  |  |  |  |  |  |
|  | Sig. (2-tailed) | 0.578 | 0.593 | 0.874 | 0.593 | 0.657 |  |  |  |  |  |  |  |  |  |
|  | R^2^ | 0.378 | 0.356 | 0.039 | 0.356 | 0.263 |  |  |  |  |  |  |  |  |  |
|  | N | 3 | 3 | 3 | 3 | 3 | 3 |  |  |  |  |  |  |  |  |
| *C. oxytoca* bacterial resistance | Pearson Correlation | 0.809^**^ | 0.945^**^ | 0.422 | 0.784^**^ | -0.281 | -0.685 | -- |  |  |  |  |  |  |  |
|  | Sig. (2-tailed) | 0 | 0 | 0.117 | 0.001 | 0.31 | 0.52 |  |  |  |  |  |  |  |  |
|  | R^2^ | 0.654 | 0.893 | 0.178 | 0.615 | 0.078 | 0.469 |  |  |  |  |  |  |  |  |
|  | N | 15 | 15 | 15 | 15 | 15 | 3 | 15 |  |  |  |  |  |  |  |
| *K. pneumoniae* bacterial resistance | Pearson Correlation | 0.04 | -0.546^*^ | -0.191 | -0.001 | 0.492 | -0.522 | -0.422 | -- |  |  |  |  |  |  |
|  | Sig. (2-tailed) | 0.888 | 0.035 | 0.495 | 0.998 | 0.062 | 0.651 | 0.117 |  |  |  |  |  |  |  |
|  | R^2^ | 0.002 | 0.298 | 0.036 | 0 | 0.242 | 0.272 | 0.178 |  |  |  |  |  |  |  |
|  | N | 15 | 15 | 15 | 15 | 15 | 3 | 15 | 15 |  |  |  |  |  |  |
| *M. morganii* bacterial resistance | Pearson Correlation | -0.297 | -0.786^**^ | -0.205 | -0.362 | 0.524^*^ | -0.796 | -0.691^**^ | 0.794^**^ | -- |  |  |  |  |  |
|  | Sig. (2-tailed) | 0.283 | 0.001 | 0.463 | 0.185 | 0.045 | 0.414 | 0.004 | 0 |  |  |  |  |  |  |
|  | R^2^ | 0.088 | 0.618 | 0.042 | 0.131 | 0.274 | 0.634 | 0.478 | 0.63 |  |  |  |  |  |  |
|  | N | 15 | 15 | 15 | 15 | 15 | 3 | 15 | 15 | 15 |  |  |  |  |  |
| *P. mirabilis* bacterial resistance | Pearson Correlation | -0.297 | -0.831^**^ | -0.294 | -0.385 | 0.645^**^ | -0.729 | -0.060^**^ | 0.750^**^ | 0.944^**^ | -- |  |  |  |  |
|  | Sig. (2-tailed) | 0.283 | 0 | 0.287 | 0.156 | 0.009 | 0.48 | 0.003 | 0.001 | 0 |  |  |  |  |  |
|  | R^2^ | 0.088 | 0.69 | 0.086 | 0.148 | 0.416 | 0.531 | 0.004 | 0.563 | 0.891 |  |  |  |  |  |
|  | N | 15 | 15 | 15 | 15 | 15 | 3 | 15 | 15 | 15 | 15 |  |  |  |  |
| *P. aeruginosa* bacterial resistance | Pearson Correlation | 0.625^*^ | 0.22 | 0.42 | 0.416 | 0.211 | -0.535 | 0.418 | 0.442 | 0.267 | 0.222 | -- |  |  |  |
|  | Sig. (2-tailed) | 0.013 | 0.43 | 0.119 | 0.123 | 0.451 | 0.641 | 0.121 | 0.099 | 0.336 | 0.427 |  |  |  |  |
|  | R^2^ | 0.391 | 0.048 | 0.176 | 0.173 | 0.044 | 0.286 | 0.175 | 0.195 | 0.071 | 0.049 |  |  |  |  |
|  | N | 15 | 15 | 15 | 15 | 15 | 3 | 15 | 15 | 15 | 15 | 15 |  |  |  |
| *S. marcescens* bacterial resistance | Pearson Correlation | 0.393 | 0.514^*^ | 0.442 | 0.609^*^ | 0.078 | -0.685 | 0.454 | -0.092 | -0.164 | -0.115 | 0.36 | -- |  |  |
|  | Sig. (2-tailed) | 0.147 | 0.05 | 0.099 | 0.016 | 0.782 | 0.52 | 0.089 | 0.745 | 0.558 | 0.682 | 0.187 |  |  |  |
|  | R^2^ | 0.154 | 0.264 | 0.195 | 0.371 | 0.006 | 0.469 | 0.206 | 0.008 | 0.027 | 0.013 | 0.13 |  |  |  |
|  | N | 15 | 15 | 15 | 15 | 15 | 3 | 15 | 15 | 15 | 15 | 15 | 15 |  |  |
| *S. aureus* bacterial resistance | Pearson Correlation | 0.685^**^ | 0.573^*^ | -0.024 | 0.51 | -0.068 | -0.583 | 0.721^**^ | -0.367 | -0.565^*^ | -0.439 | 0.195 | 0.001 | -- |  |
|  | Sig. (2-tailed) | 0.005 | 0.025 | 0.933 | 0.052 | 0.811 | 0.604 | 0.002 | 0.179 | 0.028 | 0.102 | 0.487 | 0.996 |  |  |
|  | R^2^ | 0.469 | 0.328 | 0.001 | 0.26 | 0.005 | 0.4 | 0.52 | 0.135 | 0.319 | 0.193 | 0.038 | 0 |  |  |
|  | N | 15 | 15 | 15 | 15 | 15 | 3 | 15 | 15 | 15 | 15 | 15 | 15 | 15 |  |
| *S. epidermidis* bacterial resistance | Pearson Correlation | 0.712^**^ | 0.272 | -0.275 | 0.487 | 0.124 | -0.577 | 0.443 | 0.091 | -0.255 | -0.114 | 0.243 | -0.102 | 0.814^**^ | -- |
|  | Sig. (2-tailed) | 0.003 | 0.327 | 0.322 | 0.065 | 0.659 | 0.609 | 0.098 | 0.746 | 0.36 | 0.685 | 0.382 | 0.718 | 0 |  |
|  | R^2^ | 0.507 | 0.074 | 0.076 | 0.237 | 0.015 | 0.333 | 0.196 | 0.008 | 0.065 | 0.013 | 0.059 | 0.01 | 0.663 |  |
|  | N | 15 | 15 | 15 | 15 | 15 | 3 | 15 | 15 | 15 | 15 | 15 | 15 | 15 | 15 |

***Table S10:*** *Correlation matrix, generated by SPSS, of the correlations between the bacterial resistance of analysed pathogens for clarithromycin from 2008-2022. Dark green colour and “**” indicates a significant correlation at the 0.01 level. Light green colour and “*” indicates a significant correlation at the 0.05 level. Orange colour indicates no significant correlation.*

| 10 Clarithromycin | | *S. aureus*  bacterial resistance | *S. epidermidis* bacterial resistance | *S. pneumoniae* bacterial resistance |
| --- | --- | --- | --- | --- |
| *S. aureus* | Pearson Correlation | -- |  |  |
| bacterial resistance | Sig. (2-tailed) |  |  |  |
|  | R^2^ |  |  |  |
|  | N | 15 |  |  |
| *S. epidermidis* | Pearson Correlation | 0.419 | -- |  |
| bacterial resistance | Sig. (2-tailed) | 0.12 |  |  |
|  | R^2^ | 0.176 |  |  |
|  | N | 15 | 15 |  |
| *S. pneumoniae* | Pearson Correlation | 0.012 | 0.197 | -- |
| bacterial resistance | Sig. (2-tailed) | 0.966 | 0.482 |  |
|  | R^2^ | 0 | 0.039 |  |
|  | N | 15 | 15 | 15 |

Supplemental Figures

***Fig. S1:*** *Development of bacterial resistance and DDD-prescriptions for the antibacterial drug amoxicillin from 2008 to 2022.*


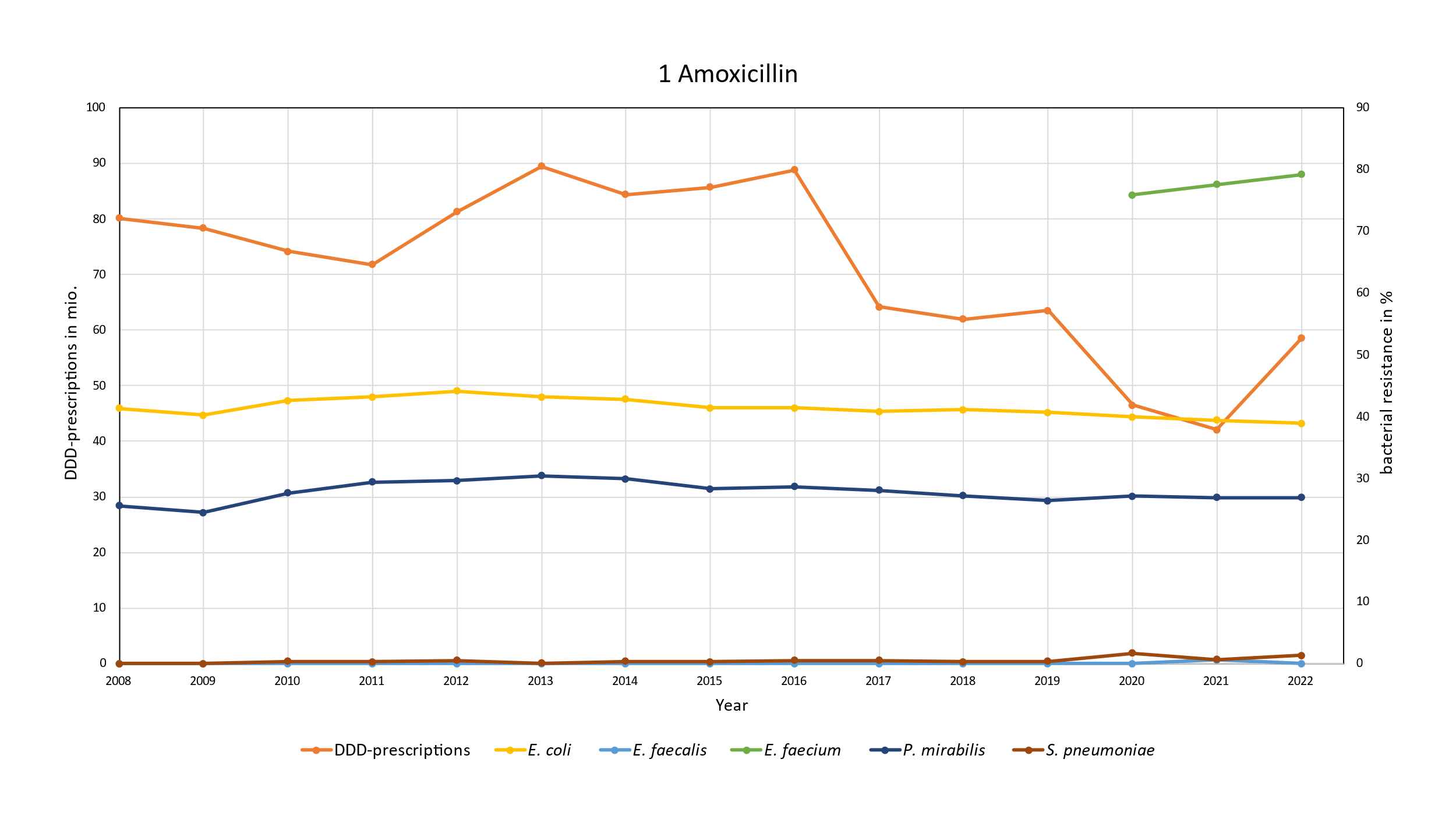


***Fig. S2:*** *Development of bacterial resistance and DDD-prescriptions for the antibacterial drug cefuroxime axetil from 2008 to 2022.*


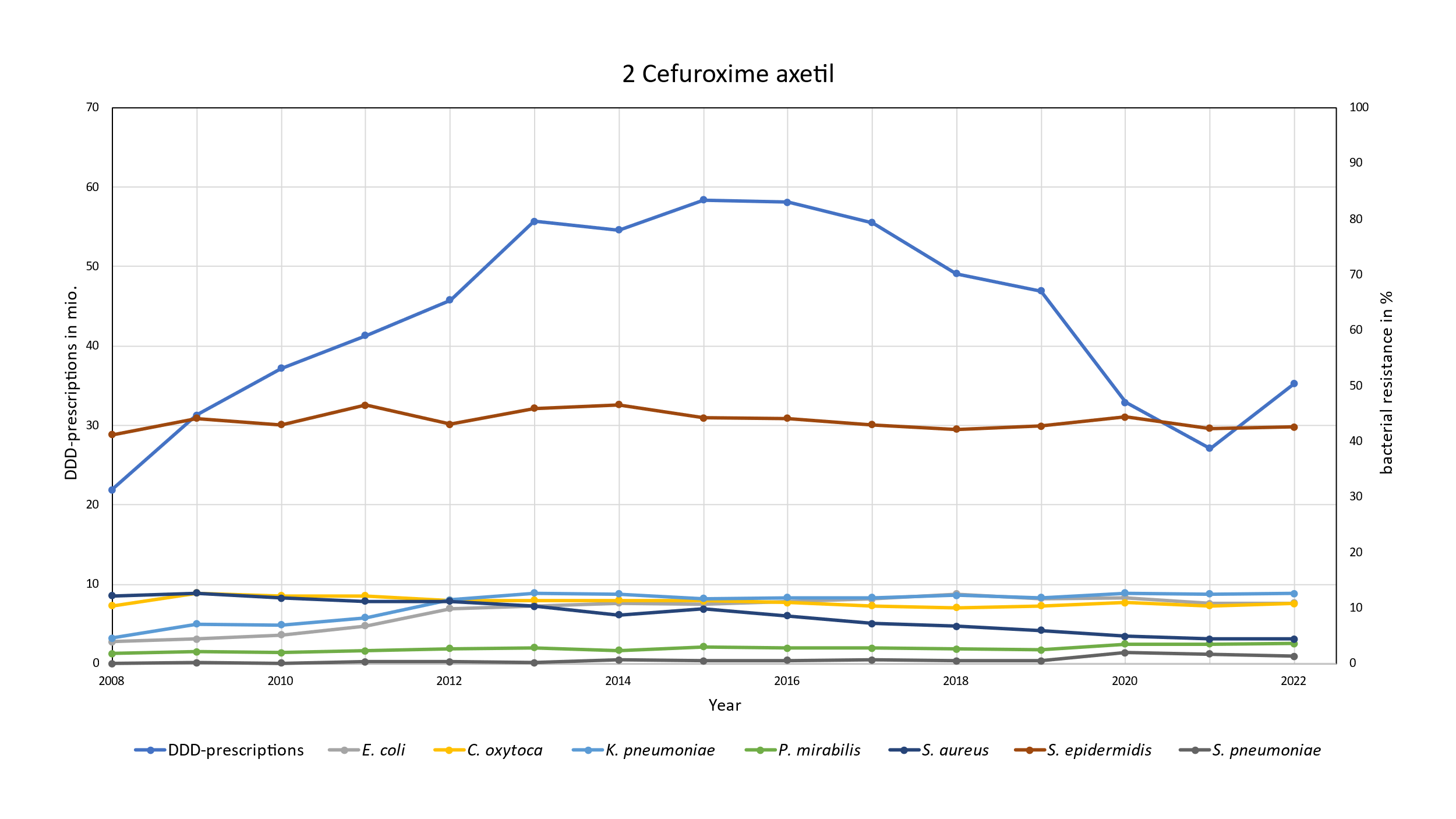


***Fig. S3:*** *Development of bacterial resistance and DDD-prescriptions for the antibacterial drug doxycycline from 2008 to 2022.*


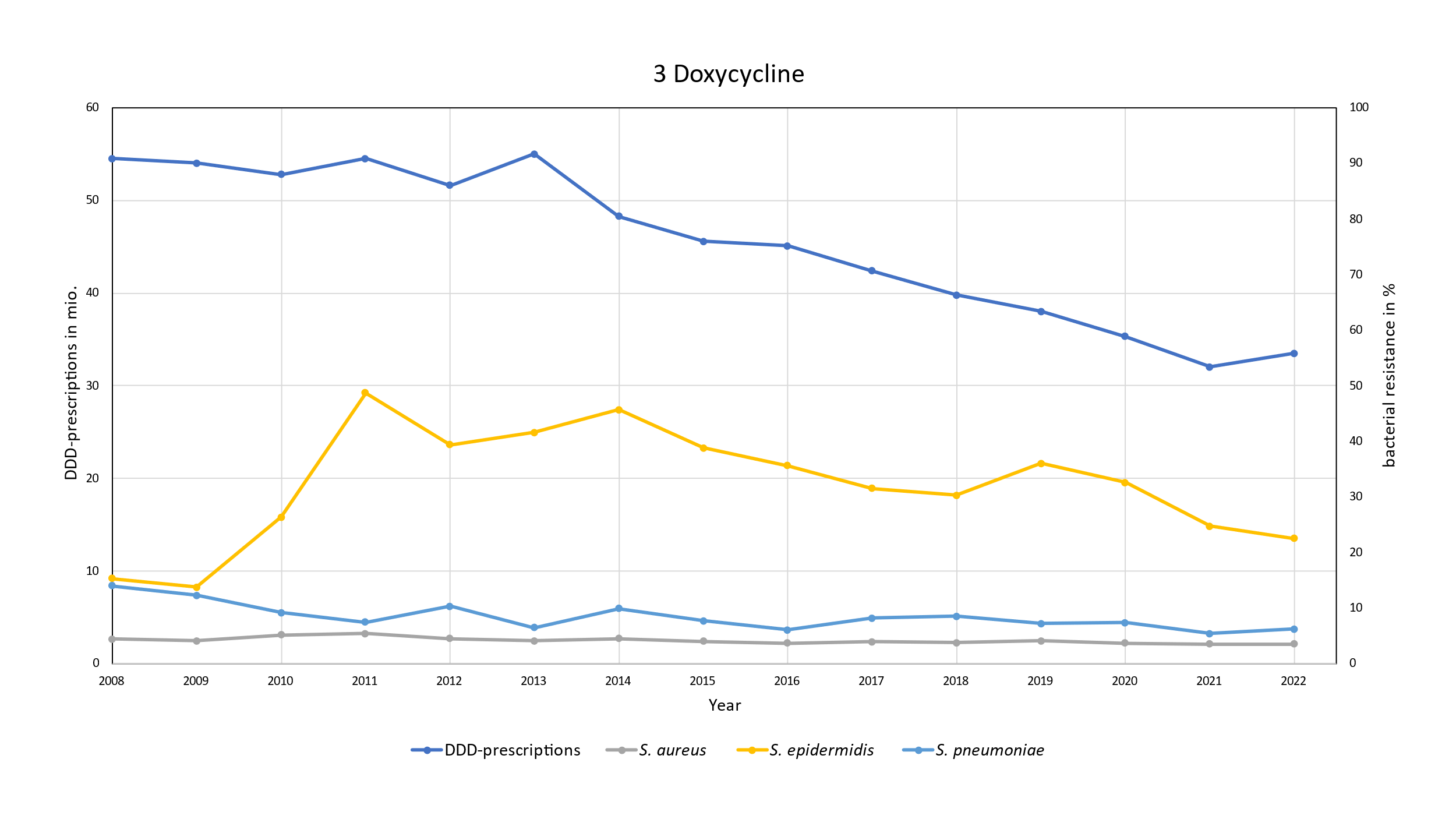


***Fig. S4:*** *Development of bacterial resistance and DDD-prescriptions for the antibacterial drug amoxicillin clavulanic acid from 2008 to 2022.*


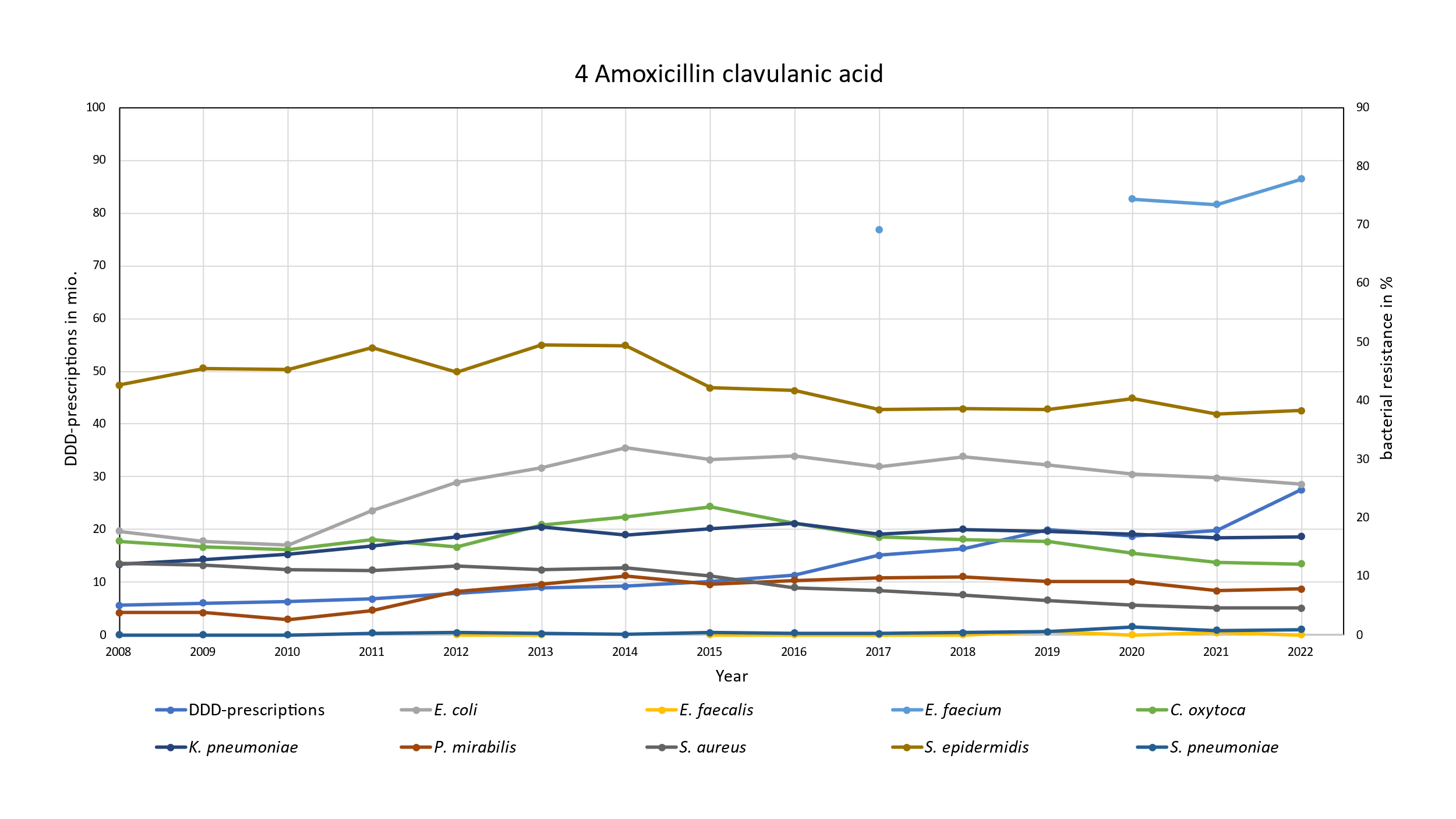


***Fig. S5:*** *Development of bacterial resistance and DDD-prescriptions for the antibacterial drug clindamycin from 2008 to 2022.*


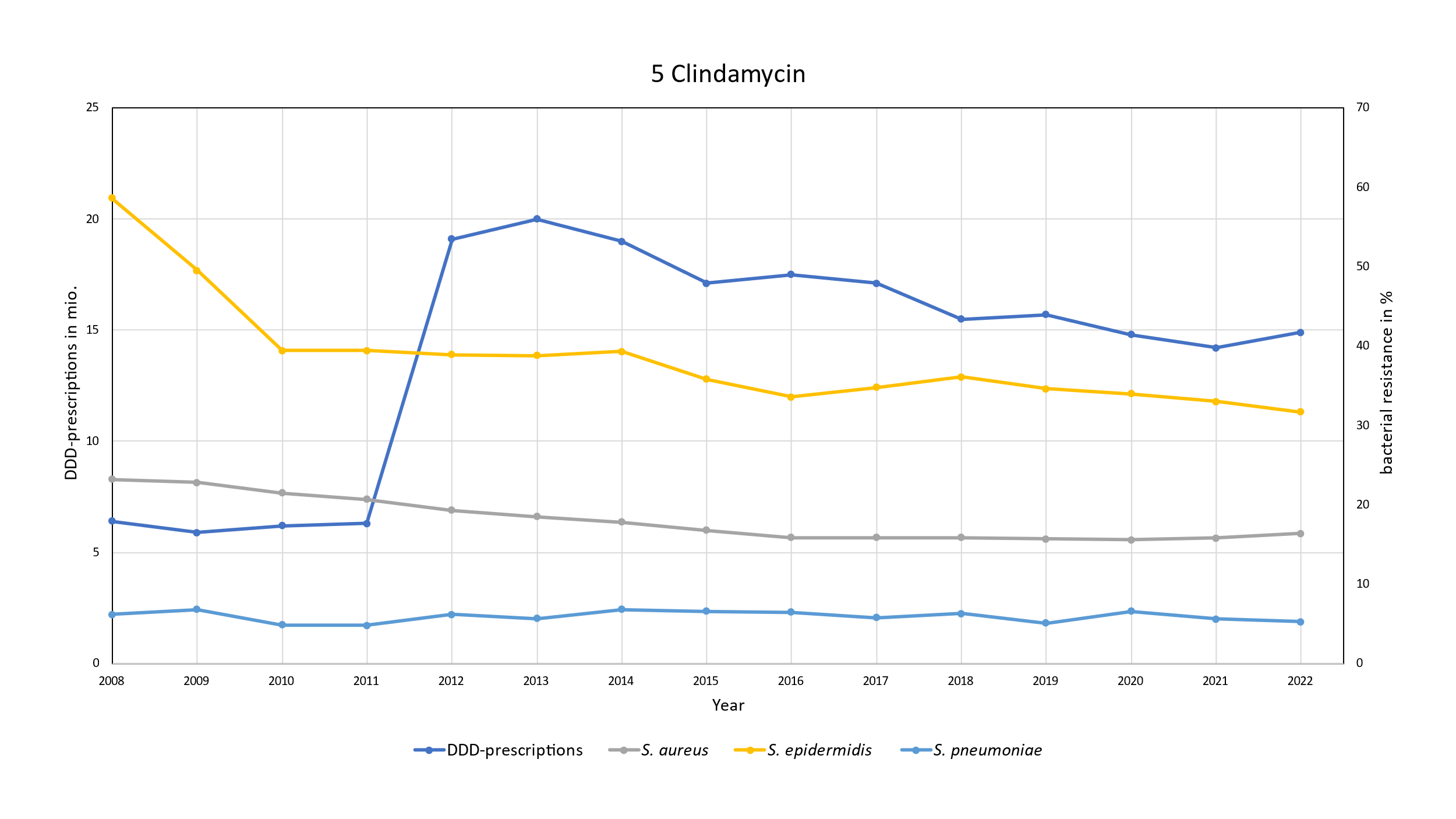


***Fig. S6:*** *Development of bacterial resistance and DDD-prescriptions for the antibacterial drug azithromycin from 2008 to 2022.*


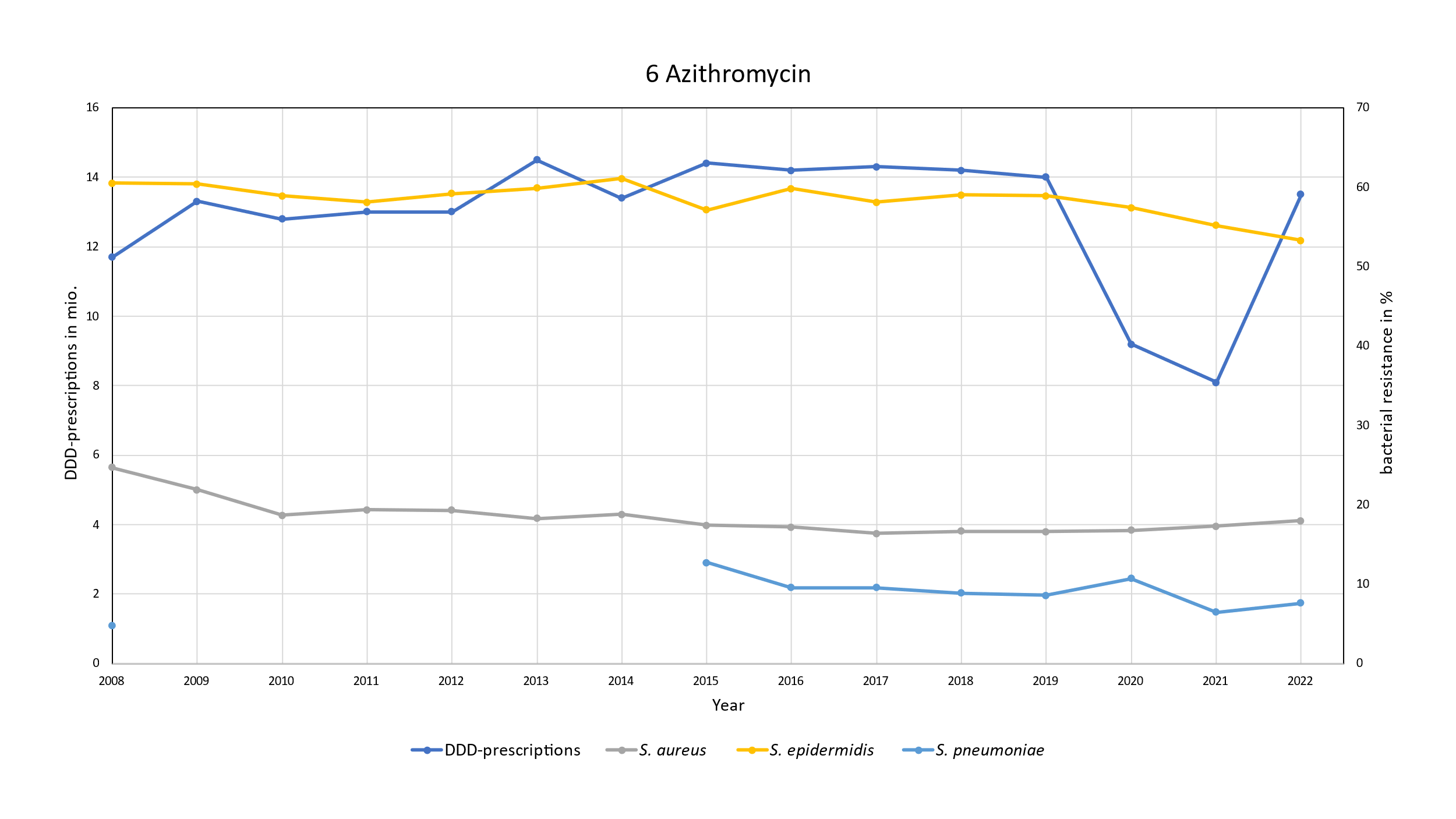


***Fig. S7:*** *Development of bacterial resistance and DDD-prescriptions for the antibacterial drug sulfamethoxazole-trimethoprim from 2008 to 2022.*


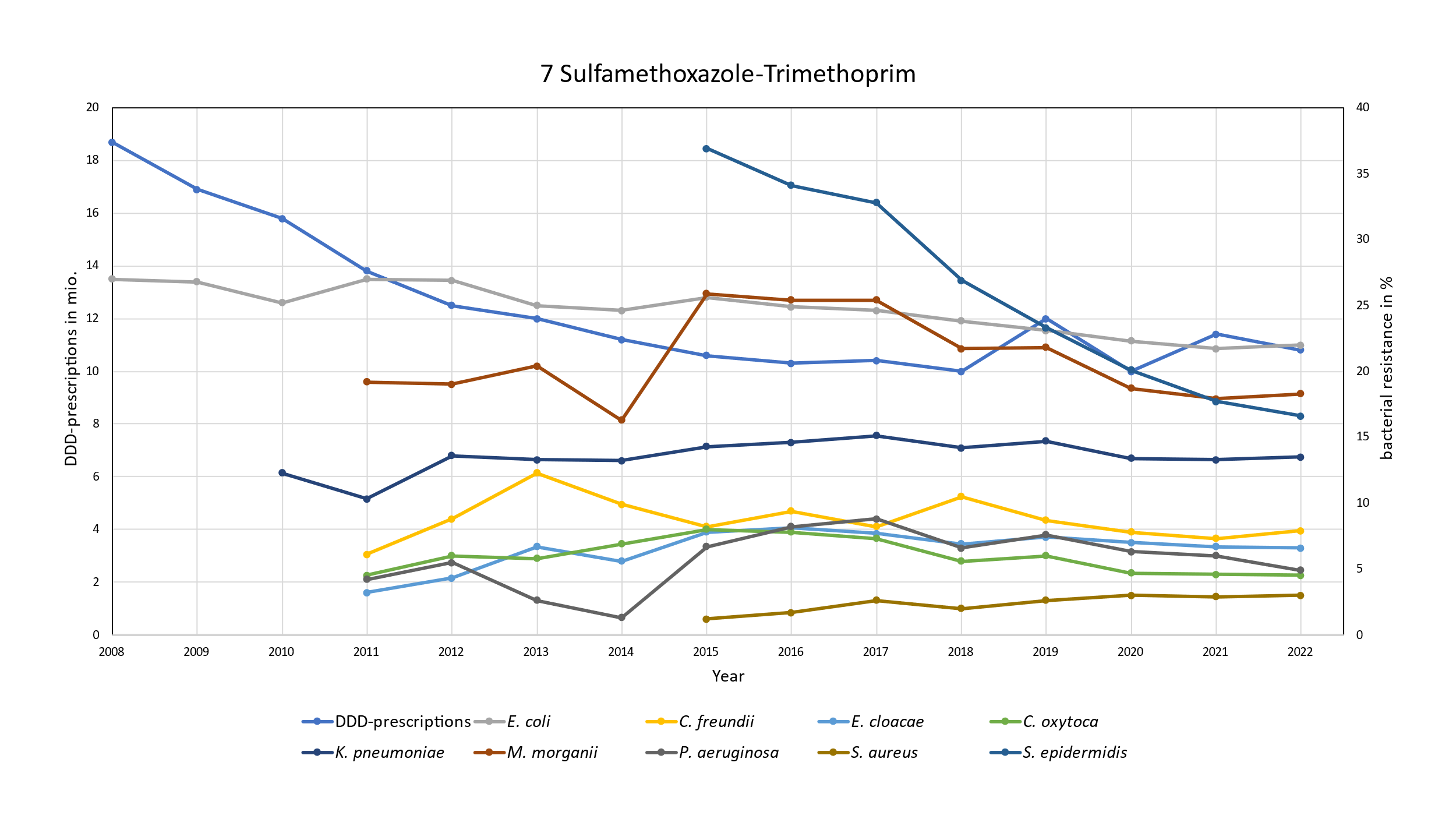


***Fig. S8:*** *Development of bacterial resistance and DDD-prescriptions for the antibacterial drug nitrofurantoin from 2008 to 2022.*


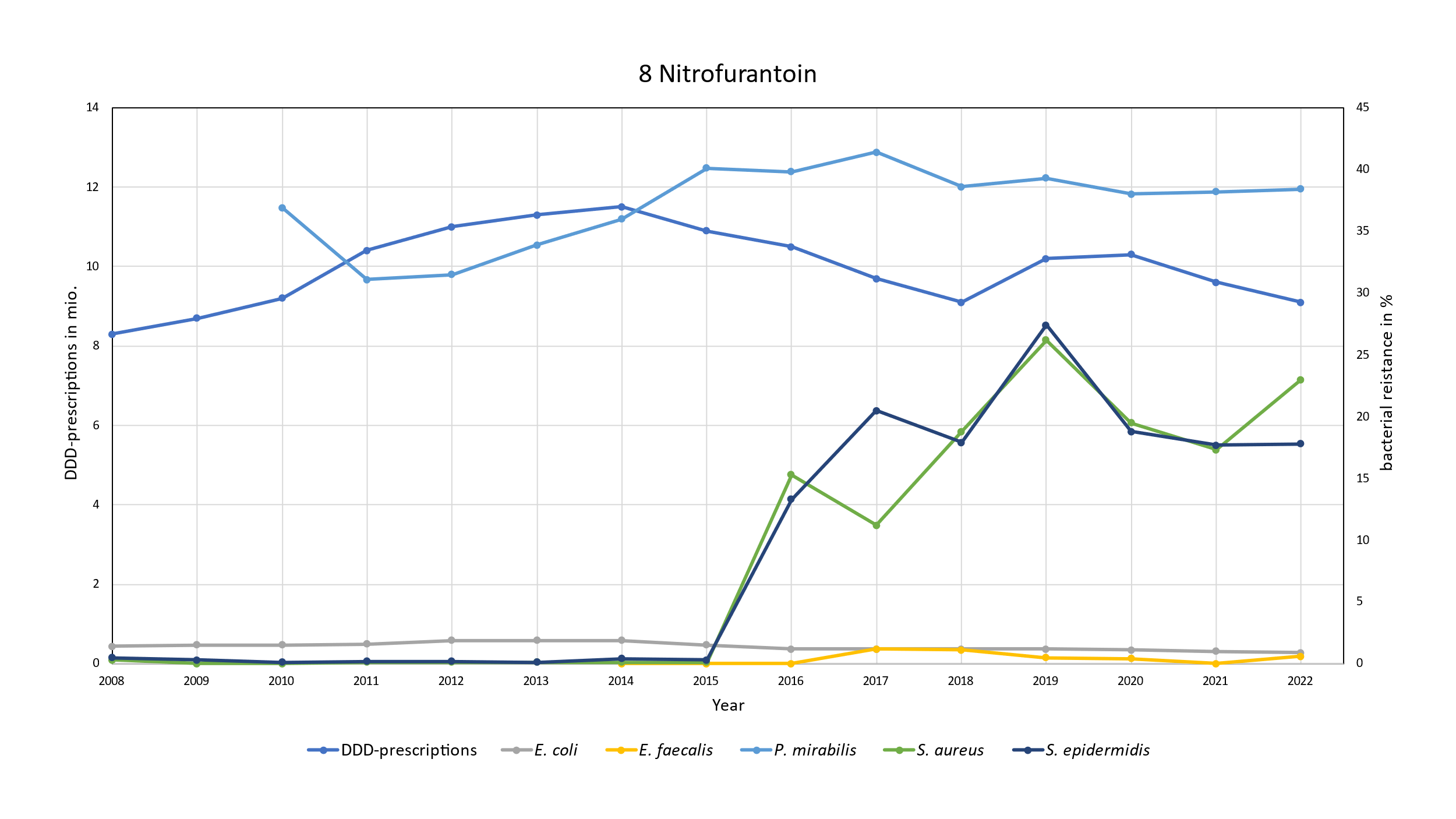


***Fig. S9:*** *Development of bacterial resistance and DDD-prescriptions for the antibacterial drug ciprofloxacin from 2008 to 2022.*


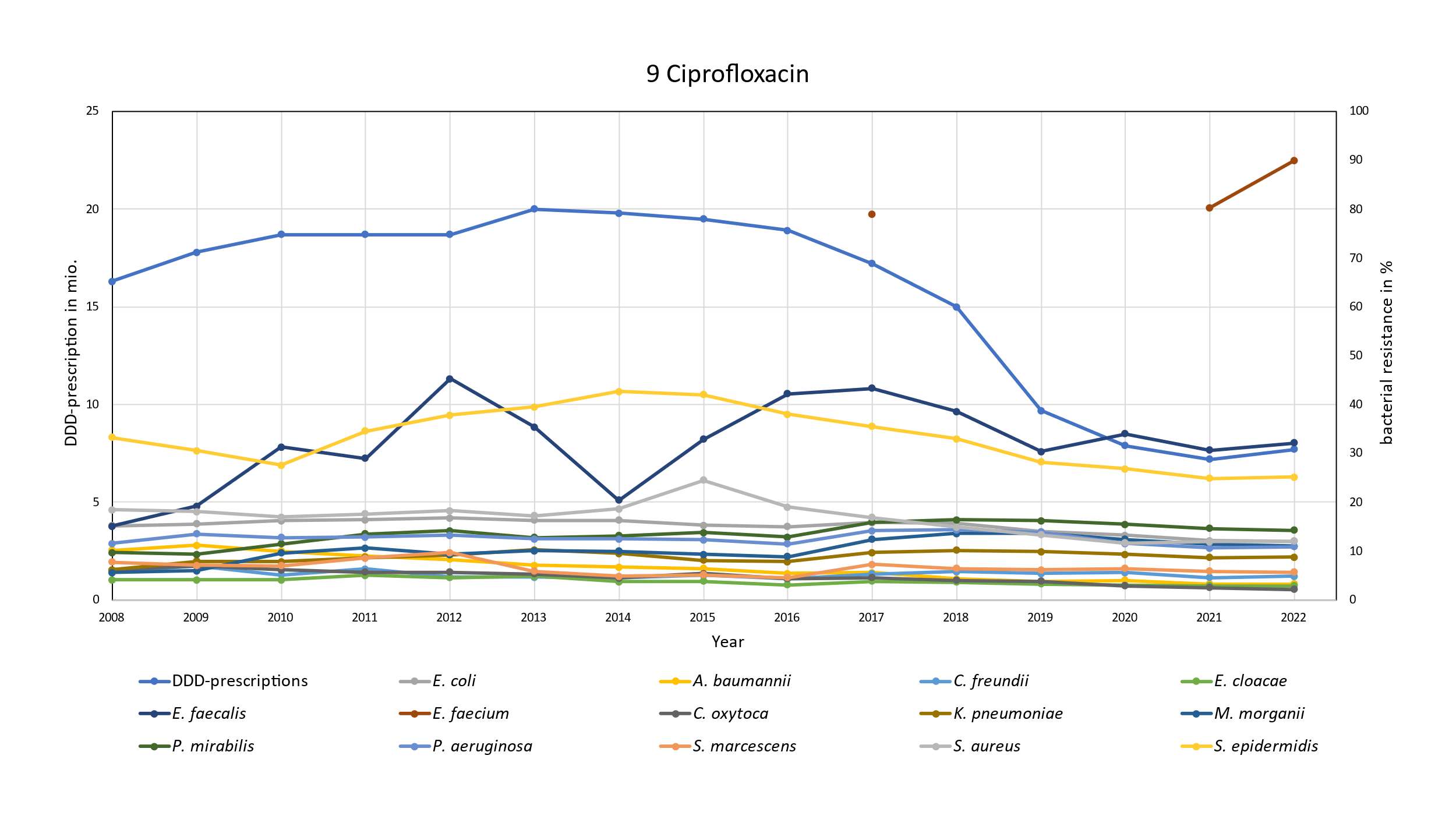


***Fig. S10:*** *Development of bacterial resistance and DDD-prescriptions for the antibacterial drug clarithromycin from 2008 to 2022.*


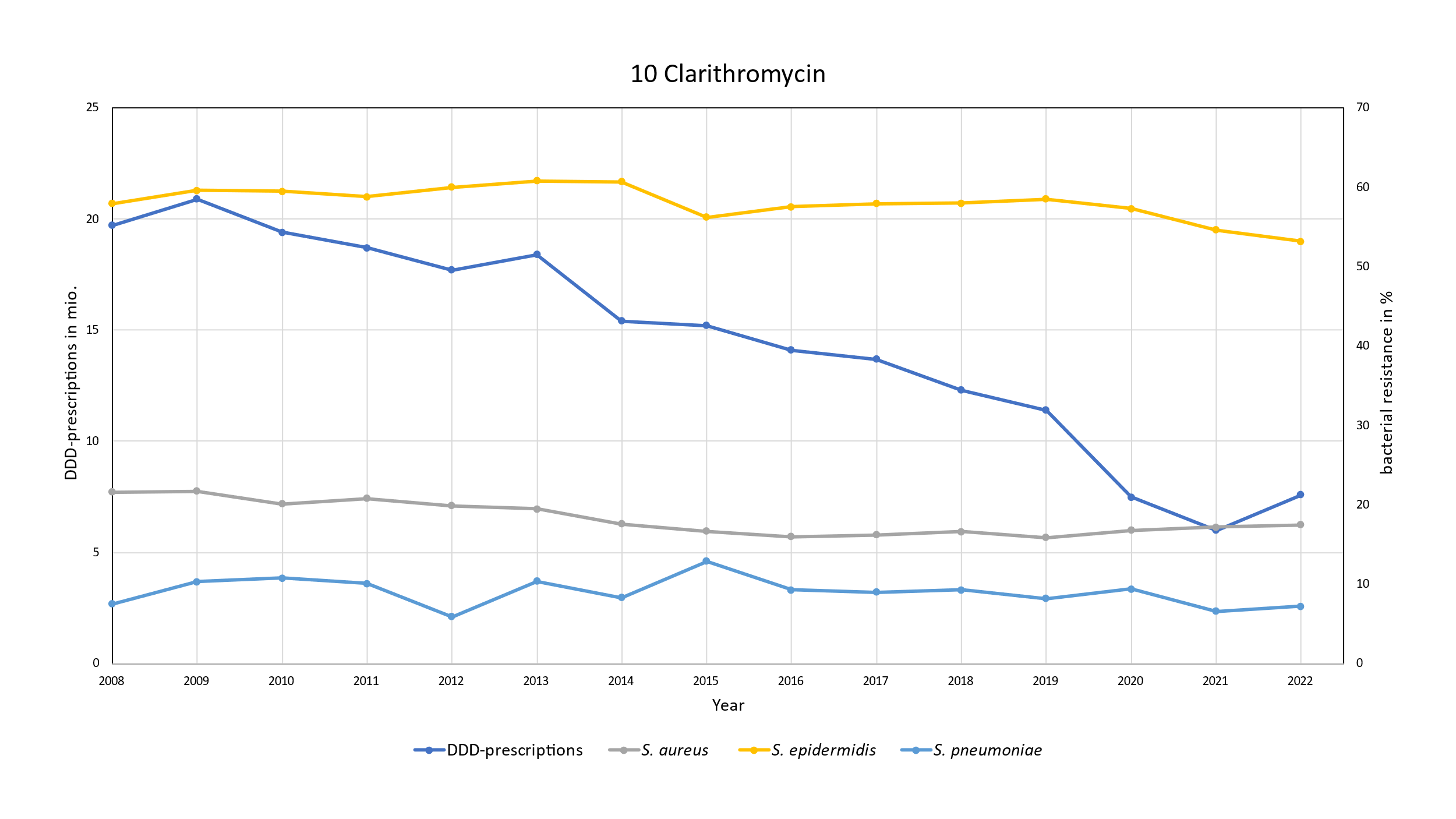


***Fig. S11:*** *Detailed information with bacterial resistance values for the best fitting antibacterial drug for treating an infection of analysed gram negative Enterobacteriaceae and its development of bacterial resistance in the analysed time period. If there was a change within the matching drug (including E. cloacae and K. pneumoniae), both antibacterial drugs and their development for the pathogen are exhibited, marked by similar colours.*


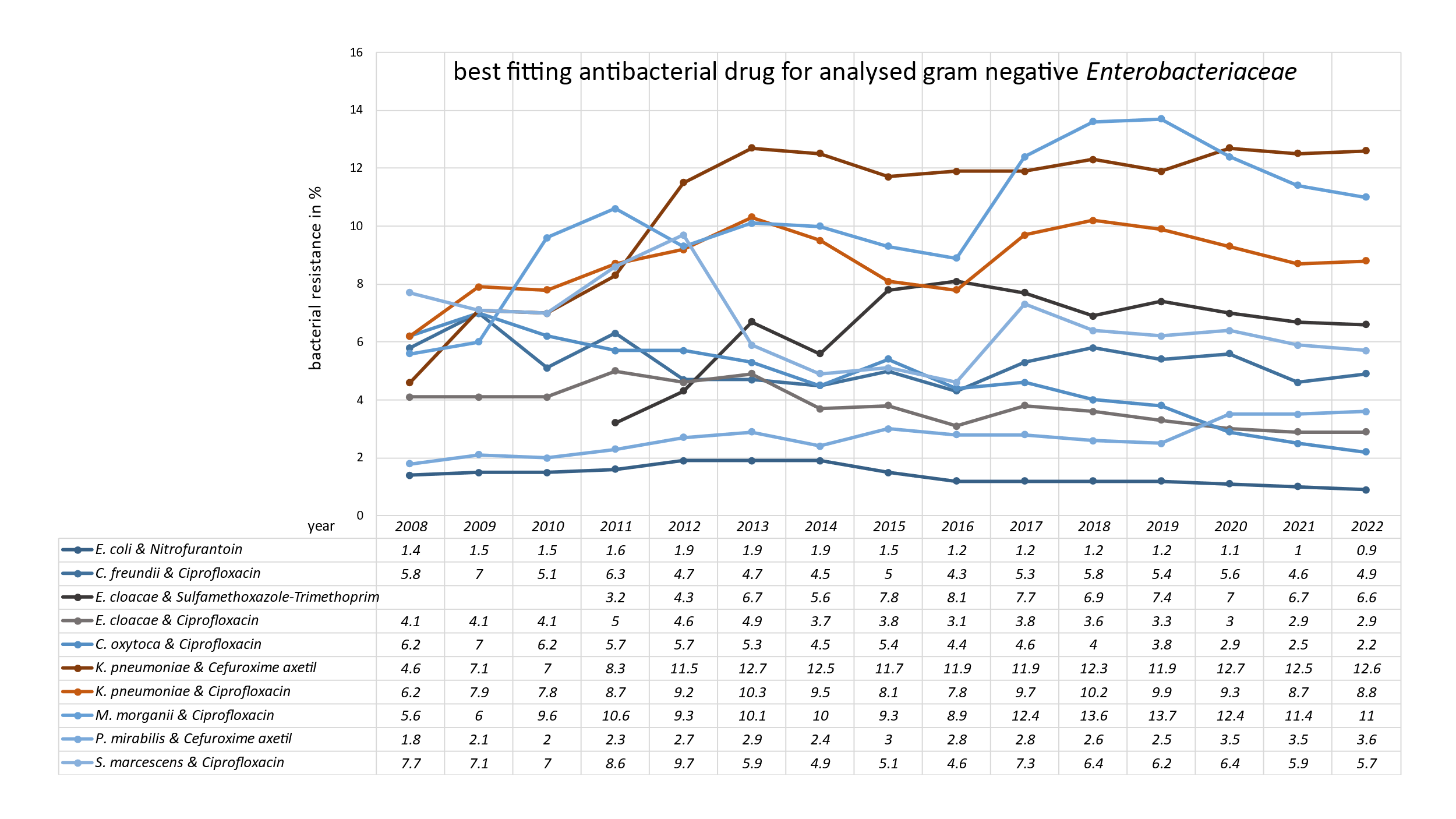


***Fig. S12:*** *Detailed information with bacterial resistance values for the best fitting antibacterial drug for treating an infection of analysed gram negative non-Enterobacteriaceae and its development of bacterial resistance in the analysed time period.*


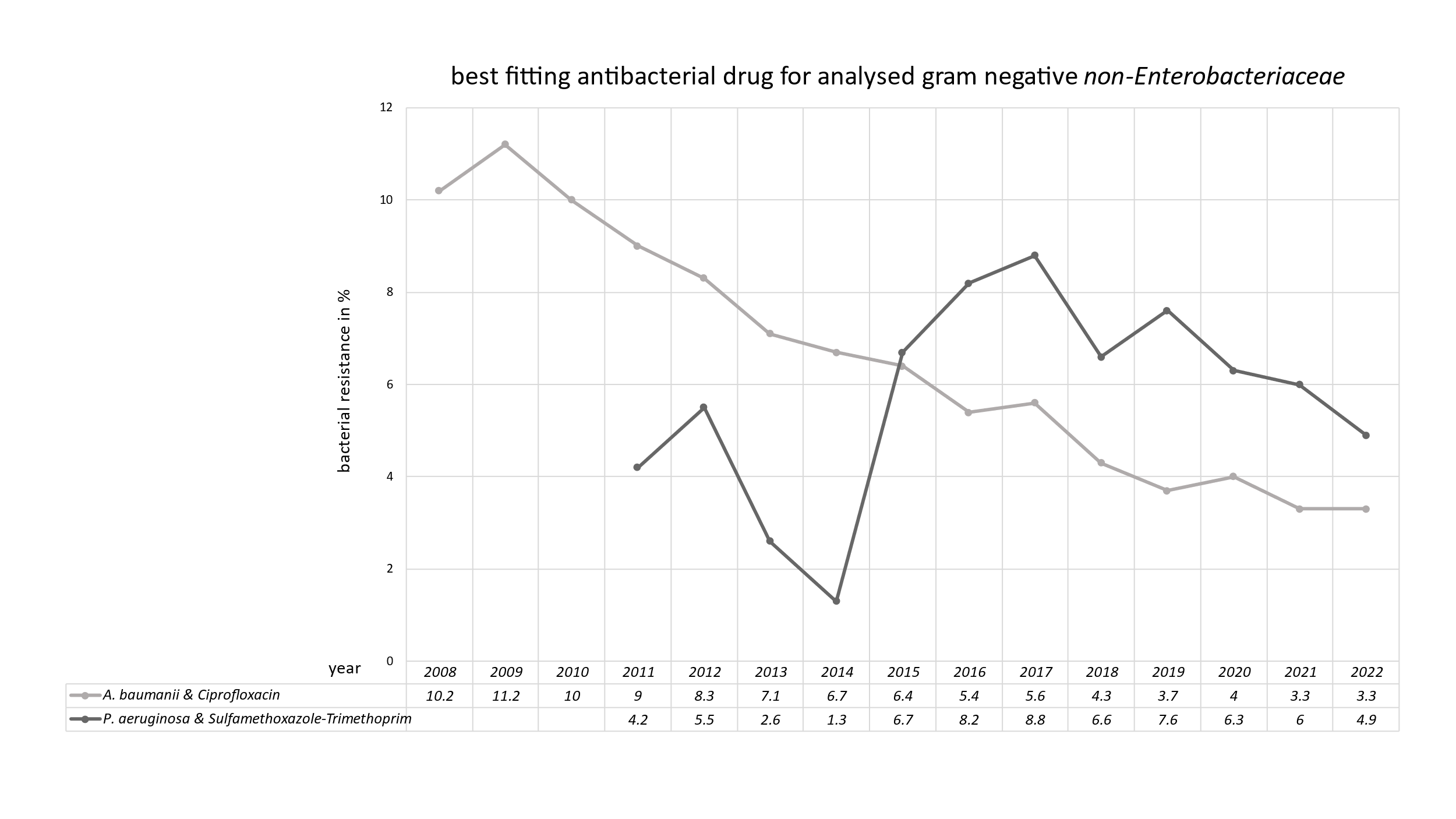


***Fig. S13:*** *Detailed information with bacterial resistance values for the best fitting antibacterial drug for treating an infection of analysed gram positive Staphylococci and its development of bacterial resistance in the analysed time period. If there was a change within the matching drug (including S. aureus and S. epidermidis), both antibacterial drugs and their development for the pathogen are exhibited, marked by similar colours.*


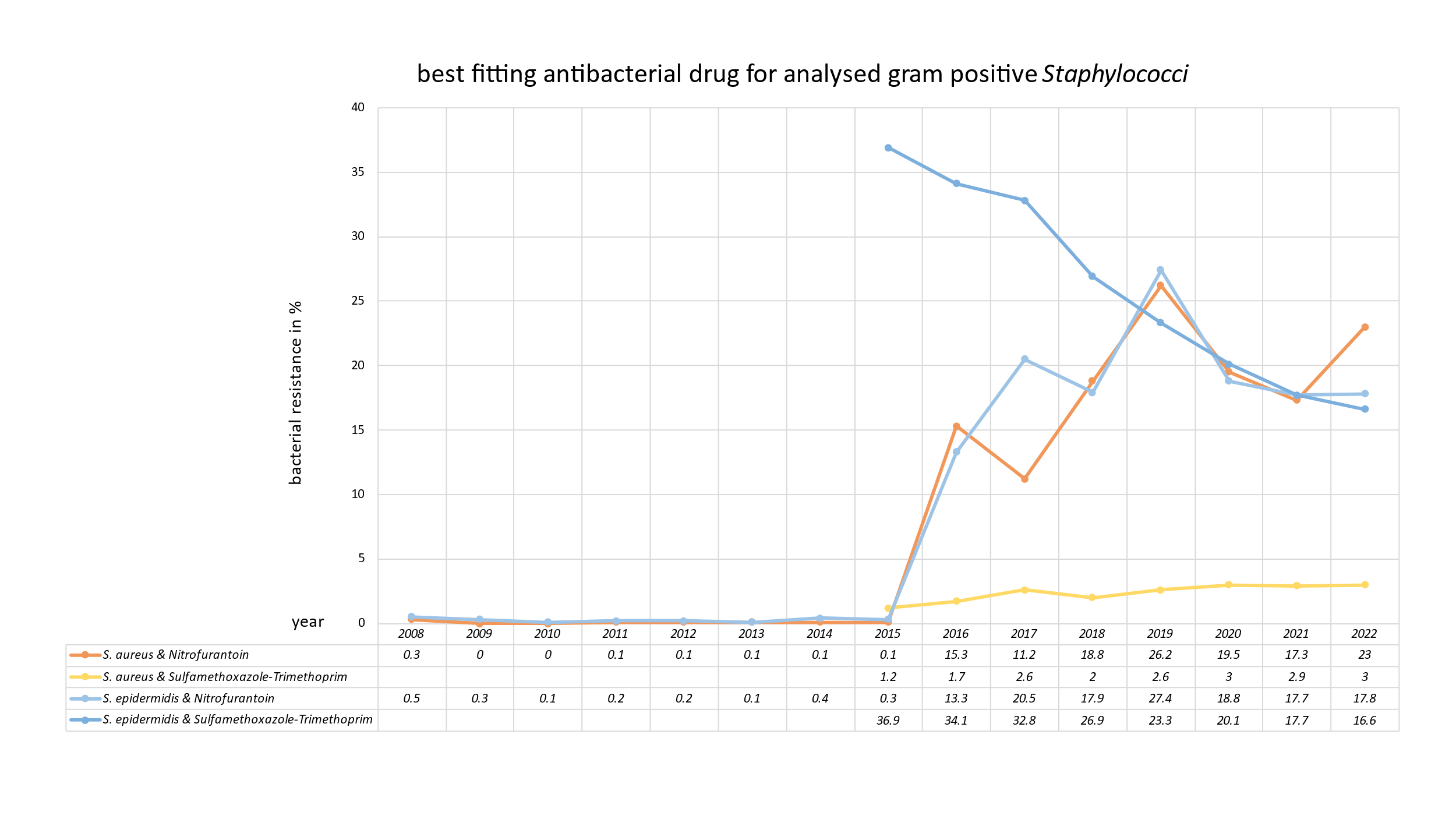


***Fig. S14:*** *Detailed information with bacterial resistance values for the best fitting antibacterial drug for treating an infection of analysed gram positive Pneumococci and its development of bacterial resistance in the analysed time period. If there was a change within the matching drug (including S. pneumoniae), both antibacterial drugs and their development for the pathogen are exhibited, marked by similar colours.*


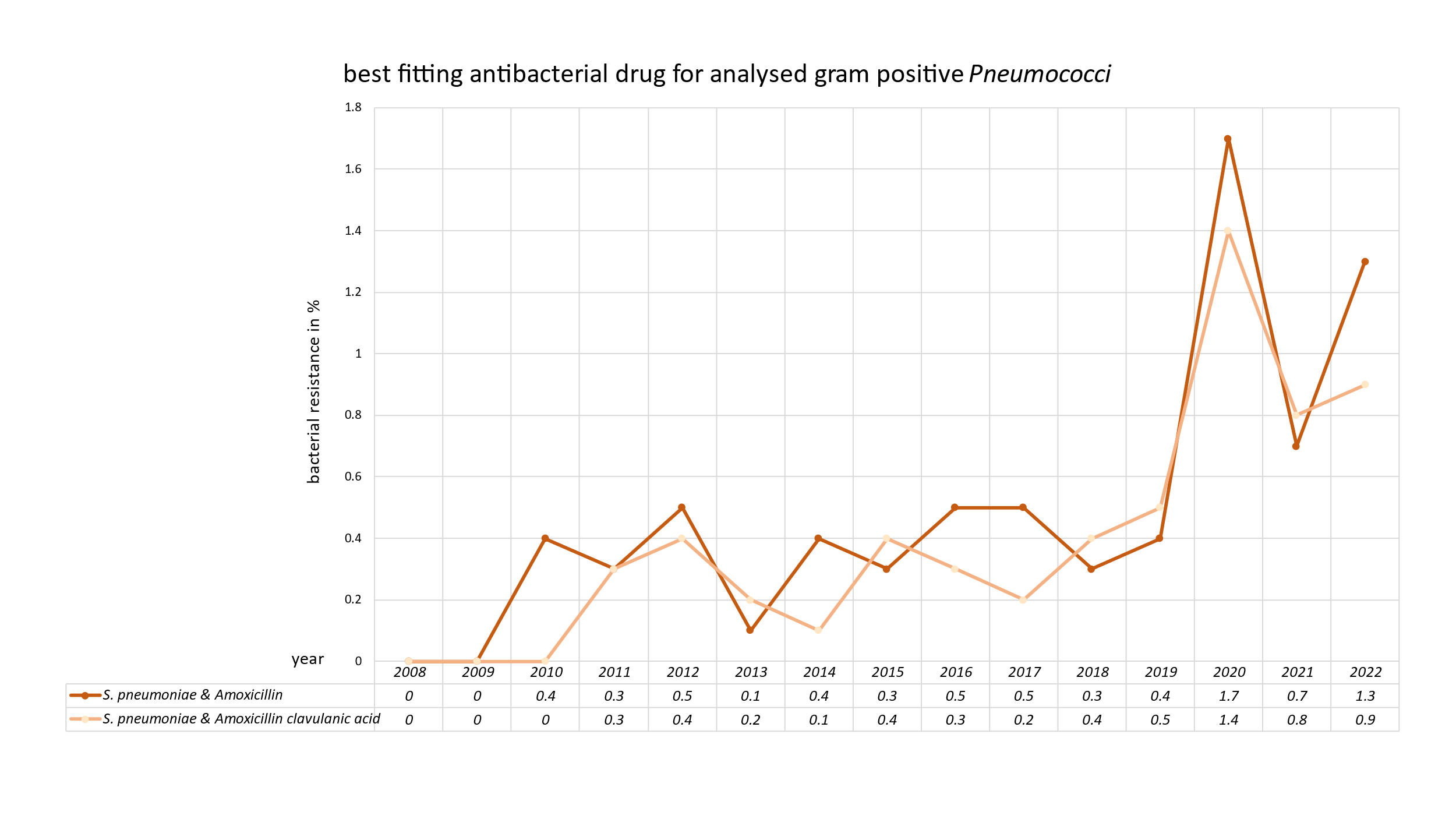


***Fig. S15:*** *Detailed information with bacterial resistance values for the best fitting antibacterial drug for treating an infection of analysed gram positive Enterococci and its development of bacterial resistance in the analysed time period.*


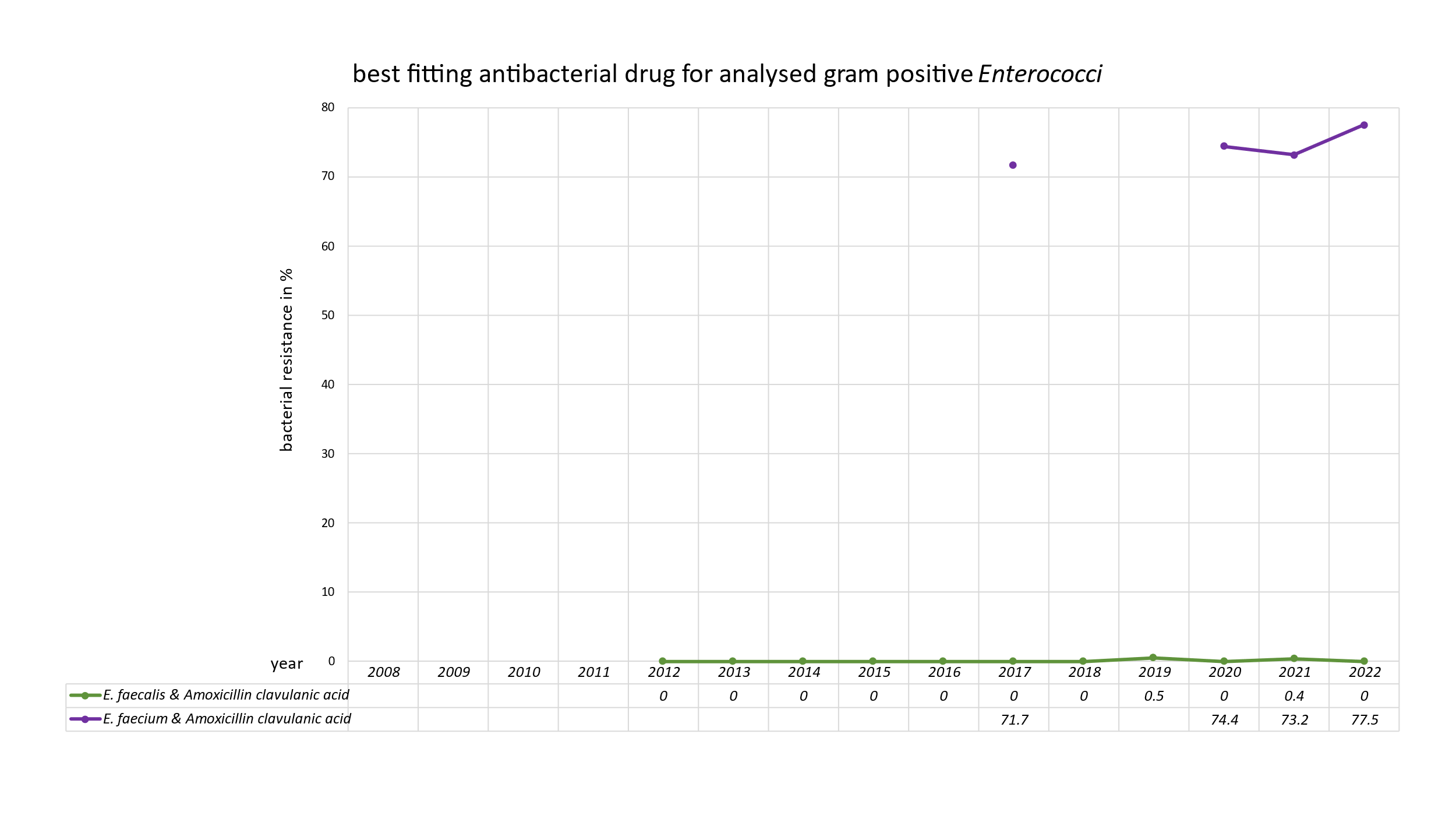

Supplement: Supplementary file 1 — Supplementary file1 (DOCX 2855 KB) [file 210_2024_3533_MOESM1_ESM.docx]
